# The Conservation and Study of Macromycetes in the Komarov Botanical Institute Basidiomycetes Culture Collection—Their Taxonomical Diversity and Biotechnological Prospects

**DOI:** 10.3390/jof9121196

**Published:** 2023-12-14

**Authors:** Nadezhda V. Psurtseva, Anna A. Kiyashko, Svetlana V. Senik, Natalya V. Shakhova, Nina V. Belova

**Affiliations:** Komarov Botanical Institute of the Russian Academy of Sciences, 197376 St. Petersburg, Russia; anna.kiyashko@binran.ru (A.A.K.); senik@binran.ru (S.V.S.); nshakhova@binran.ru (N.V.S.); nbelova@binran.ru (N.V.B.)

**Keywords:** fungal biodiversity, culture collections, fungal strains, enzymatic activity, oxidases, fungal lipids, secondary metabolites

## Abstract

Culture collections (CCs) play an important role in the ex situ conservation of biological material and maintaining species and strains, which can be used for scientific and practical purposes. The Komarov Botanical Institute Basidiomycetes Culture Collection (LE-BIN) preserves a large number of original dikaryon strains of various taxonomical and ecological groups of fungi from different geographical regions. Started in the late 1950s for the investigation of Basidiomycetes’ biological activity, today, in Russia, it has become a unique specialized macromycetes collection, preserving 3680 strains from 776 species of fungi. The Collection’s development is aimed at ex situ conservation of fungal diversity, with an emphasis on preserving rare and endangered species, ectomycorrhizal fungi, and strains useful for biotechnology and medicine. The main methods applied in the collection for maintaining and working with cultures are described, and the results are presented. Some problems for the isolation and cultivation of species are discussed. The taxonomical structure and variety of the strains in the collection fund are analyzed, and they show that the taxonomical diversity of fungi in the LE-BIN is commensurable with the largest CCs in the world. The achievements from the ex situ conservation of the diversity of macromycetes and the main results from the screening and investigation of the collection’s strains demonstrate that a number of strains can be prospective producers of enzymes (oxidoreductases and proteases), lipids, and biologically active compounds (terpenoids, phthalides, etc.) for biotechnology and medicine.

## 1. Introduction

There are two complementary approaches to fungal conservation: in situ (on-site) and ex situ (off-site). In situ conservation means protecting fungi in their natural habitat together with their associated plants. This generally implies protecting natural habitats themselves so that fungal populations are maintained in their natural ecotopes. Ex situ conservation achieves protection by removing a living sample of the species from its natural ecological context, growing it on a nutrition medium, and maintaining it in pure culture [1,2]. Culture collections (CCs), genetic resource collections, and biological resource centers play a key role in the ex situ conservation of fungi [2,3,4]. The main coordinators of work on fungal conservation ex situ are the World Federation for Culture Collections (WFCC, https://wfcc.info/, accessed on 20 September 2023) and the European Culture Collections’ Organisation (ECCO, https://www.eccosite.org/, accessed 20 September 2023), which bring together collections and users, direct activities, and exchange information and technologies. At the time of writing (September 2023), 843 CCs from 79 countries are currently registered in the World Data Centre for Microorganisms (WDCM, https://ccinfo.wdcm.org/statistics, accessed on 20 September 2023). Altogether, the CCs preserve 3,473,147 microorganisms, of which 896,096 are fungi. The majority of large collections preserve various types of organisms, e.g., bacteria, fungi, yeasts, algae, archaea, and cell lines, whereas much fewer CCs specialize in the conservation of species diversity and strain variety of a specific type of organisms, in particular, fungi (the largest is CBS, The Netherlands), and only a few collections preserve only macromycetes (e.g., CCBAS, the Czech Republic; TFC, Estonia; CCDBM, Korea; IBK, Ukraine; FIB, the Republic of Belarus). The Komarov Botanical Institute Basidiomycetes Culture Collection (LE-BIN, Russia) is one of only a few specialized collections maintaining only species of basidial and marsupial macromycetes (i.e., fungi with visible (by the naked eye) sexual reproduction structures), which are also usually called mushrooms or macrofungi.

The LE-BIN was established at the Komarov Botanical Institute in the late 1950s (the first strain dates back to 1956) as the research institute’s culture collection. Basidiomycetes cultures were studied as a source of food and feed proteins, enzymes, and biologically active compounds, mainly proteolytic enzymes with thrombolytic and milk-clotting activity, oxidoreductases with laccase and peroxidase activities, polysaccharides with anti-tumor activity, and secondary metabolites (sterols and hallucinogenic compounds). Until the mid-1990s, the collection had rapidly increased the variety of strains related to a particular species: the producers of biologically active compounds that were objects of study during those years. Since then, a new direction in the development of the collection has started, following the increasing need to preserve biodiversity on the planet and the world trend in fungal conservation [1]. The collection’s development plan was modified to include ex situ conservation of the taxonomical and ecological diversity of basidiomycetes, with emphases on preserving rare and endangered species in Russia, maintaining ectomycorrhizal fungi, and culturing species useful for biotechnology and medicine. The diversity of marsupial macrofungi has been cultured and maintained in the collection only since the beginning of this century. The objective was to preserve, ex situ, and study, in culture, as many different macromycete species as possible in this process, including the collection of fungi and their isolation in pure culture, the identification of natural specimens and verification of isolates, and the cultivation and investigation of strains. Currently, the species diversity of basidiomycetes preserved in the LE-BIN is comparable with the largest collections in the world, such as the ATCC (USA), CBS (The Netherlands), the MUCL (Belgium), the DSMZ (Germany), and some others. 

The purpose of this article is to present the main trends in working with the LE-BIN cultures, to analyze the taxonomical structure and variety of the strain in the Culture Collection fund, and to show the achievements from the ex situ conservation of macromycetes’ diversity and the main results from the screening and investigation of the collection’s strains, which can be prospective producers of enzymes and biologically active compounds for biotechnology and medicine.

## 2. Materials and Methods Applied in the LE-BIN Collection

### 2.1. Origination and Isolation of Fungi

The majority of the LE-BIN Culture Collection strains are original isolates from various regions of Russia (areas of the European part, the Caucasus, the Urals, Siberia, and the Far East), mainly from protected zones, i.e., nature reserves and national parks, but there are many strains from other countries (Vietnam, the USA, Finland, Ukraine, etc.). The Collection staff manually obtain fungal isolates during annual field work from collected fruiting bodies using their tissues or spores. The culturing usually takes place in field stations, rarely in specially equipped tents, or sometimes directly in an open area. The whole process of obtaining pure cultures is undertaken with the maximum level of cleanliness and aseptic conditions that could be achieved during the field trips. Portable UV sterilizers help to create clean working spaces and the required conditions for culturing. Sterile plastic Petri dishes (Ø 35–40 mm) filled with sterilized (121 °C, 15 min) beer-wort or malt extract (4%), agar (2%), and water solution of antibiotic (0.06% kanamycin or gentamicin) were used for the isolation process; the tools were burned in the flame of an alcoholic lamp before every use. When the first signs of mycelium growth or spore germination appeared, a small piece of growing culture was moved into 2 mL sterile plastic vial (cryovials) with 0.7 mL of the same medium as in the Petri dishes for further growth and transportation. This allows for better protection of isolated material until its cultivation and study in laboratory conditions. 

In addition to the original cultures, the collection preserves strains obtained on the basis of exchange from other domestic and foreign collections.

### 2.2. Preservation Methods

Preservation of strains in the LE-BIN collection is carried out in parallel by three methods: (1) Subculturing in tubes on beer-wort agar slants in a refrigerator at 4–6 °C (14–16 °C for tropical strains); (2) disk method in screw-cap cryovials under distilled water at 4–6 °C (14–16 °C for tropical strains); (3) cryopreservation method in screw-cap cryovials in 10% aqueous glycerol solution at −80 °C, with a freezing rate of 1 °C min^−1^.

### 2.3. Strains Verification Methods

#### 2.3.1. Cultivation Media

The following agar media are usually used for cultivation of the LE-BIN strains: beer-wort agar (BWA)—4% beer-wort from various breweries (St. Petersburg, Russia) and agar 20 g/L (Difco, Sparks, MD, USA); malt extract agar (MEA)—50 g/L (Oxoid, Basingstoke, Hampshire, England) or 35 g/L (Conda, Madrid, Spain); potato dextrose agar (PDA)—39 g/L (Panreac, Darmstadt, Germany) or 15 g/L of commercial potato flakes, glucose 10 g/L (Vecton, St. Petersburg, Russia), and agar 20 g/L (Difco, Sparks, MD, USA). Basic liquid media for inoculum growth and for both stationary and submerged cultivation: 4% or 1% beer-wort (BW); malt extract (ME)—15.0 g/L (Conda, Madrid, Spain); glucose–peptone (GP) medium of the following composition (g/L): 3.0 peptone, 10.0 glucose, 0.6 KH_2_PO_4_, 0.4 K_2_HPO_4_, 0.5 MgSO_4_, 7H_2_O, 0.05 CaCl_2_, 0.01 ZnSO_4_, and 0.005 FeSO_4_; pH-5.6–6.0 before sterilization. Sterilization for 30 min at 121 °C. 

#### 2.3.2. Morphological Methods

The morphology of colonies was described following Stalpers’ diagnostic characters and terminology [5]. Macromorphology: (a) advancing zone and the outline of the colony; (b) the texture of the mycelial mat; (c) the colony color; (d) the odor; and (e) the reversum (reverse side) of the colony. Since a culture medium affects the texture and color of the colony, two standard culture media, MEA and PDA, were usually used to study the culture characteristics. Micromorphology: (a) presence of clamps; (b) width and (c) type of hyphae; (d) presence of various hyphal structures, like anastomoses, hyphal rings, hyphal strands, rhizomorphs and sclerotia, cystidia and gloeocystidia, encrusted hyphae, crystals, hyphal nodes, various swellings, etc.; and (e) the presence of propagative structures: arthroconidia, conidia, chlamydospores, blastoconidia, etc. Sometimes, these structures have taxonomic significance [5,6]. It should be noted that Clemenson’s classification is more complex and unclearly structured; however, it more fully takes into account the characteristics of the hyphal systems of different species. The colony morphology was examined after 2 and 4 weeks. Micromorphology was studied under Zeiss Axio Imager A1 and Axio Scope A1 (Zeiss, Oberkochen, Germany) using transmitted light and UV light.

#### 2.3.3. Molecular Methods

Verification of the strains was performed by PCR analysis of the nuclear ITS (and rarely LSU) rDNA region (fungal bar code), sampling a small piece of mycelium from the advancing zone of the colonies and using Thermo Scientific Phire Plant Direct PCR Kit (Vilnius, Lithuania) and standard basidiomycetes primers ITS1F and ITS4B [7] for amplification. Primers ITS1F and ITS4 were applied for heterobasidiomycetes and marsupial macromycetes. Primers LROR and LR5 were used for LSU (https://www2.clarku.edu/faculty/dhibbett/protocols_folder/primers/primers.pdf, accessed on 20 September 2023). In some cases, DNA was extracted using the Nucleo Spin Plant II (Macherey-Nagel, Duren, Germany) or similar kits. Sequencing was performed with an ABI model 3130 Genetic Analyzer (Applied Biosystems, Foster City, CA, USA) using the BigDyeTM Terminator Cycle Sequencing Ready Reaction Kit (AB) at the Core Facility Centre of the Komarov Botanical Institute RAS. Raw data and the final sequences were processed using MEGA 7—MEGA XI [8,9]. A search for closely related sequences was performed using the BLAST program in the NCBI public database (https://www.ncbi.nlm.nih.gov, accessed on 4 December 2023). Generated sequences were deposited to the GenBank NCBI.

#### 2.3.4. Preparing of Substrates for Fruiting in Culture

Fruiting in culture is a reliable method of confirming the authenticity of strains. Some species can fruit spontaneously in culture, directly in tubes on agar slants, or in Petri dishes, but strains are usually grown on sawdust to induce fruiting. *Betula* (birch) sawdust and wheat bran (3:1, respectively) were mixed, and boiling water was added until the substrate became moistened. Glass beakers or other autoclavable containers (300–500 mL) were filled with the prepared substrate, covered with aluminum foil and autoclaved at 121 °C for 1 h. After cooling, the substrate was inoculated with diced strains grown in Petri dishes on MEA and incubated in darkness at room temperature until the substrate was fully covered with mycelium; then, beakers (containers) were transferred to a growth chamber (Sanyo, Osaka, Japan, 20 °C, 2000 lx, 90% humidity). The covers were removed when the cultures began to form visible primordia.

### 2.4. Growth and Enzymatic Activity Methods

#### 2.4.1. Lineal Growth in Petri Dishes

Inoculum plugs (7 mm in diameter) were placed mycelium-side down on the edge of 90 mm (60 mm for slow-growing strains) Petri dishes containing BWA or MEA and incubated for 6 weeks in growth chambers in the dark at 25 °C to evaluate the growth rate of the strains. The experiments were conducted in three replicates on each medium. The growth rate was recorded every other day (or every day in case of fast-growing species), and linear mycelial extension (mm) and standard deviation were estimated using MS Excel 2016 statistics tool.

#### 2.4.2. Cultivation on Liquid Media

Inoculum for cultivation on liquid media was grown in 1 L Erlenmeyer flasks with porcelain beads on the BW or GP medium (100 mL) at 25 °C, stationary, for 10–14 days, depending on the growth rate of the fungus. Before inoculation, the inoculum was crushed with porcelain beads at 180 rpm for 20 min to form a homogeneous suspension, which was added to the cultivation flasks with the BW or GP medium to a final volume of 10% under sterile conditions. Submerged cultivation was performed in a shaker incubator Innova R44 (New Brunswick, Enfield, CT, USA) in 750 mL conical flasks, 100–200 mL media or BioSan ES-20 (Riga, Latvia) in 250 mL conical flasks, and 50 mL media at 180 rpm for 15–20 days depending on the biomass production and glucose consumption. Culture liquid sampling to measure strain growth indicators (pH, biomass production, glucose consumption, enzymatic activity, etc.) was performed daily or less often, depending on the growth capacity of the strains.

#### 2.4.3. Express Method for Oxidative Enzymes

To qualitatively evaluate the activity of oxidoreductases (ligninolytic enszymes) by the express method [10], strains were grown in the dark at 25 °C in Petri dishes (90 mm) on BWA medium. For inoculation, a plug (Ø 7 mm) of a 7-day-old mycelium was placed on the edge of a Petri dish with the mycelium side down. Oxidase activity was determined after two weeks of growth. Mycelial plugs (3 replicates on each substrate) with a diameter of 7 mm were cut at the edge of the growing colony and transferred to the wells of microbiological plates, followed by a dropwise addition of guaiacol (2 mL/100 mL H_2_O) (Sigma, St. Louis, MO, USA) and 1.0% syringaldazine (Sigma, St. Louis, MO, USA) in C_2_H_5_OH. The activity was evaluated visually by color reaction intensity on a scale from “–” (no activity) to “+ + +” (very high activity) at intervals of 5, 15, 30, and 60 min and 3 and 24 h.

#### 2.4.4. Enzymatic Activity by Application Tests

Application tests were carried out in Petri dishes with related sterilized (121 °C, 30 min) agar substrates. 

Oxidoreductases were estimated using widespread substrate ABTS (2,2′-azino-bis 3-ethylbenzothiazoline-6-sulfonic acid, Sigma, St. Louis, MO, USA) [11] and agar (Difco, Sparks, NV, USA) in distilled water in concentrations of 0.55 and 20.0 g/L, respectively. Optionally, 15.0 g/L malt extract (Conda, Madrid, Spain) could also be added in case the observation of the activity development is to be carried out on the dynamics of the fungal growth. Three mycelial plugs of 7 mm in diameter were cut near the edge of a growing colony and evenly applied on the surface of the Petri dish filled with substrate (in the presence of ME—one plug on the center of the plate). The plates were incubated in the growing chamber at 25 °C for 48 h (2 wks if ME was added). Positive activities were evaluated by the diameter of colored zones (or by the difference between the diameter of colored zones and diameter of colonies if ME was added to the substrate) measured in two mutually perpendicular directions. The following criteria were used to determine the intensity of the oxidative enzymatic reaction (on ABTS-agar substrate): weakly positive—zone around the inoculums is less than 10 mm in diameter; positive—zone around the inoculums is 11–20 mm in diameter; strongly positive— zone around the inoculums is more than 25 mm in diameter.

A dye decolorization assay of polyphenolic dye azure B, indicating the presence of degradation potential in basidiomycetes, was performed in Petri dishes with a diameter of 90 mm. The strains for the inoculum were grown in Petri dishes (Ø 60 mm) on BWA in the growing chamber at 25 °C for 2 weeks. Then, mycelial blocks (7 mm in diameter) from the marginal zone of the actively growing colony were put in the center of new Petri dishes (Ø 90 mm) with mycelial layer downwards on MEA medium containing azure B (Sigma, St. Louis, MO, USA) at a concentration of 75 mg/L. The plates with mycelial plugs were incubated at 25 °C. The degradation potential of the strains was determined by measuring the diameter of the dye discoloration zones (in two mutually perpendicular directions) formed around the inoculum every 7 days of cultivation for 4 weeks. Petri dishes (Ø 90 mm) containing MEA with azure B under the same concentration with no fungal inoculum and incubated under the same conditions served as a control. The degradation potential of the investigated fungi was evaluated by time (days) and intensity (diameter, mm) of dye discoloration in Petri dishes.

Proteolytic activity was estimated using fibrin film as substrate (fibrinolytic activity). Fibrinolytic activity was assessed using a modified express method [12] based on the area of lysis zones of fibrin film. Best results in achieving stablity at 37 °C fibrin films were obtained using standard fibrinogen preparations (without plasminogen admixture) and thrombin produced by the Kaunas Factory of Bacterial Drugs (Kaunas, Lithuania). Additionally, 9 mL of 0.3% fibrinogen and 0.3 mL of 1% thrombin in saline (pH 7) were accurately mixed, transferred into a Petri dish (Ø 90 mm), and left until the fibrin polymerized. Mycelium plugs, 0.3 mL of culture filtrate, or the test enzyme solution (protein 1 mg/mL) were applied to the surface of the film. Three replicates were placed in one plate. The plates were incubated at 37 °C for 18 h; afterward, the zones of fibrin lysis were measured in two mutually perpendicular directions. Fibrinolytic activity was expressed in mm or in units per 1 mL of the test solution. The zone of fibrin plate lysis of 10 mm^2^ was taken as one conventional unit.

The other substrate for proteolytic activity was gelatin (gelatinase activity) consisting of sterilized distilled water and 4% food gelatin heated in the microwave until dissolved. Application, incubation, and zone measurements were carried out as described above (25 °C for 48 h). In case of high activity, the time of incubation was shortened up to 24 h.

Cellulolytic activity was estimated using distilled water substrate consisting of (g/L) 10.0 micro crystal cellulose (Chemapol, Praha, Czech Republic or Vecton, St. Petersburg, Russia) and 20.0 agar (Difco, Sparks, MD, USA). Application and incubation were carried out as described above (25 °C for 48 h). The activity of cellulases was determined by the presence of an enlightened zone around the inoculum (diameter, mm). For zone development, the surface of plates was short-term treated with solution of 0.5% I in 2% of KI. The same parameters were used to record the intensity of the reaction as in the determination of oxidoreductase activity.

Each activity was assessed in three replicates. The average diameter of zones (mm) and standard deviation (*n* = 6) were estimated using the MS Excel 2016 statistics tool.

#### 2.4.5. Analysis of Lipids

The composition of fatty acids was studied by gas chromatography–mass spectrometry as described in the publication of Kotlova et al. [13], and phospholipid profiles were analyzed by ESI-MS as described in [14].

#### 2.4.6. Analysis of Secondary Metabolites

Methods of metabolomic analysis and study of some secondary metabolites (phthalides, sparassol, and other orsellinic acid derivatives) of the LE-BIN strains were described in recent publications [15,16,17]. Triterpenoids were converted into their trimethylsilyl (TMS) ethers by reaction with N,O-bis(trimethylsilyl)trifluoroacetamide (Sigma-Aldrich, St. Louis, MO, USA) and analyzed by GC-MS using the gas chromatograph Agilent 7820A with MSD 5975 [18].

### 2.5. Taxonomical Analysis

The taxonomic position of the collection’s strains is given mainly according to He et al. [19], Wijatawardene et al. [20], and Kalichman et al. [21] for Agaricales, taking into account a number of modern publications concerning some taxonomic groups [22,23,24,25,26,27,28,29,30].

## 3. Results and Discussion

### 3.1. Ex Situ Conservation of Macromycetes Diversity

If in situ conservation is the more evident event, aiming to conserve fungi in their natural habitat together with their associated plants, then ex situ conservation is usually treated as a supplemental approach. Nevertheless, human impacts on the environment create so many threats to biodiversity, including habitat loss resulting from the conversion of forests to farmlands, urbanization, pollution, etc., that have a negative effect on species survival that sometimes ex situ conservation may become the only option to save them from elimination. However, the most vivid benefit of ex situ long-term conservation is the possibility of using the diversity of fungi and multiplying their genetic resources for scientific, industrial, agricultural, and medicinal purposes. Ex situ fungal conservation is the most reliable to realize in specialized culture collections (CC), genetic resource collections (GRC), and biological resource centers (BRC).

**Figure 1:**
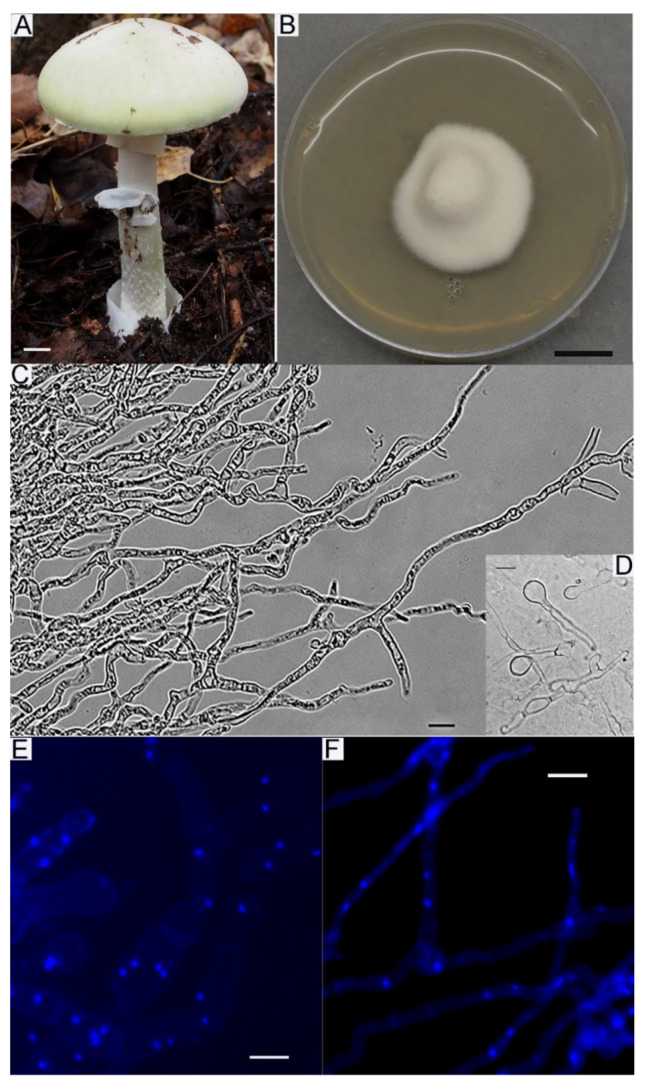
*Amanita phalloides* LE-BIN 4016. (**A**) Natural basidioma. (**B**) Colony mat on MEA. Scale bars are 10 mm. (**C**–**F**) Micromorphology: (**C**)—hyphal system, (**D**)—swellings on hypha, (**E**,**F**)—dapi-stained nuclei glow under UV light indicating dikariotic hypha. Scale bars are 10 µm.

The success of obtaining pure macromycete cultures depends on many factors. Unfortunately, not all fungi can be cultured easily. The main “uncultured” group is ectomycorrhizal fungi (EMF). The majority of EMF species are problematic for isolation in culture, and even when cultures are obtained successfully, they grow very slowly, often forming so-called “colonies of restricted growth” when they reach 20–30 mm in diameter and then stop their growth. Nevertheless, a large number of EMF species were isolated in culture, and their growth and morphological and cultural characteristics were studied [31,32,33], as well as the influence of nutrient media components on the growth and development of EMF cultures [34,35,36]. Success has also been achieved in obtaining a pure culture and cultivation of “uncultured” species such as *Entoloma clypeatum* (L.) P. Kumm. [37], *Cantharellus cibarius* Fr. [38,39], *C. anzutake* W. Ogawa, N. Endo, M. Fukuda & A. Yamada [40,41] and *Tricholoma matsutake* (S. Ito & S. Imai) Singer [42]. Maintaining EMF in culture is a part of the LE-BIN collection development plan. Our experience showed that only *Suillus* species from EMF could be relatively easily cultured on standard malt extract media. LE-BIN culture collection conserved ex situ several *Suillus* species (i.e., *S. variegatus* (Sw.) Richon & Roze, *Suillus grevillei* (Klotzsch) Singer, *S. americanus* (Peck) Snell, *S. spraguei* (Berk. & M.A. Curtis) Kuntze and *S. asiaticus* (Singer) Kretzer & T.D. Bruns). However, the isolation and cultivation of *Amanita phalloides* (Vaill. ex Fr.) Link proved to be the most difficult. This culture was obtained in 2013 during a special expedition to the Voronezh region (Russia), with the goal of isolating *A. phalloides* in culture. Over 100 basidiomata were collected, and about half of the youngest and freshest of them were used in attempting to obtain a culture. Eventually, two pure isolates were obtained, one of which died soon after, and the other (strain LE-BIN 4016, Genbank accession number MW036159) is still alive and maintained in the collection (Figure 1). Cultures of several other EMF species were successfully obtained in the LE-BIN (*Amanita muscaria*, *Boletus edulis*, *Leccinum aurantiacum*, *Lactarius torminosus*, *L. deliciosus*, and some others), but they lost viability after several years of being maintained in the collection. Besides EMF, some species of soil saprotrophic and xylotrophic fungi (e.g., certain species of *Mycena*, *Volvariella*, *Pluteus*, *Tricholomopsis*) are problematic for obtaining pure cultures. The success in culturing does not only depend on the ecology or taxonomy of fungi. The weather and time passed from specimen collection and culturing conditions may influence the process of pure culture isolation.

**Figure 2:**
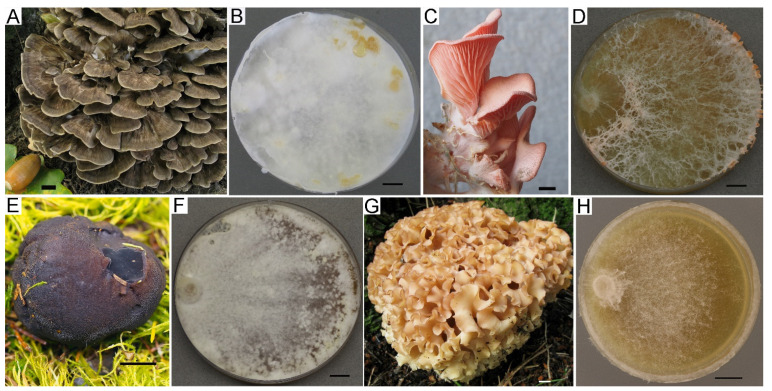
Natural basidiomata (voucher specimens) and colony mats of fungi from Russian Red Book. (**A**,**B**) *Grifola frondosa*. (**C**,**D**) *Pleurotus djamour*. (**E**,**F**) *Sarcosoma globosum*. (**G**,**H**) *Sparassis crispa*. Scale bars are 10 mm.

Preserving rare and endangered species is the other priority of fungal ex situ conservation in the LE-BIN collection. Currently, the fund contains strains of 94 species protected in Russia at the regional level and 6 species included in the Red Book of the Russian Federation in 2008 [43], i.e., *Ganoderma lucidum* s. lat. (26 strains), *Grifola frondosa* (Dicks.) Gray (7 strains), *Hericium flagellum* (Scop.) Pers. (4 strains), *Pleurotus djamor* (Rumph. ex Fr.) Boedijn (5 strains), *Sarcosoma globosum* (Schmidel) Casp. (3 strains), and *Sparassis crispa* (Wulfen) Fr. (3 strains). Several examples of fungi that are protected in Russia and their colony mats are presented in Figure 2.

Increased attention in the LE-BIN collection is paid to the ex situ conservation of fungi species with medicinal properties and biotechnological potential. It is well known that macromycetes are an inexhaustible source of useful products. Their nutritional, medicinal, and biotechnological values are very high. Fungi produce a variety of health-promoting compounds such as amino acids, proteins, dietary fiber, and polysaccharides, which open a wide range of options for using mushrooms for food and medicinal purposes. These include fresh fruiting bodies, their processed products (frozen, dried, irradiated), and additives in other products (baked goods, drinks, flour, chips, fast food, sauces, and even candies) [44]. In modern medicine, mushrooms are of interest as a source of many biologically active compounds, including polysaccharides, terpenoids, steroids, lectins, statins, polyphenols, phenols, alkaloids, antibiotics, etc., which may enhance immune function or have antitumor, antioxidant, antiviral, antibacterial, and other activities. Compounds such as lignocellulosic enzymes, lectins, proteases, and protease inhibitors have been used for the development of new drugs [45,46]. Investigation of the biotechnological potential of the LE-BIN collection strains will be covered below in the relevant sections.

The aim of fungal preservation in CC is to maintain purity, viability, and genomic integrity, avoid the selection of variants, and lessen the prospects of strain deterioration for their successful application [47]. Methods of preservation play a very important role in the successful achievement of this aim. The preservation of each strain in the collection should be carried out using no fewer than three methods in parallel. In this publication, we will discuss methods based on our experience in the preservation of the LE-BIN strains. Preservation using malt agar slants is the basic method. This method has been applied to all strains in the collection, although it is the most labor-consuming method, requiring subculturing periodically from 0.5 to 1 year for strains preserved in tubes with cotton plugs and 5–7 years for strains covered by plastic autoclavable plugs. It is difficult to maintain the viability of EMF during the long-term preservation. It was noticed that regardless of the tube plugs used, *Suillus* strains should be subcultured at least once a year, whereas the *A. phalloides* strain requires subculturing every 3–4 months. Unfortunately, when this method is applied for long-term strain preservation, it sooner or later causes strain deterioration [48]. This well-known problem is encountered not only when fungal strains are maintained in culture collections but also when they are used as long-term producers in biotechnology [49].

The disk method in screw-cap cryovials under distilled water at 4–6 °C was suggested by Burdsall and Dorworth in 1994 for wood-decaying fungi. Mycelial plugs under distilled water were supposed to be stored at room temperature for over 7 years [50], but later, cold storage in sterile water was proven to achieve better results in terms of strains surviving during long-term preservation compared to room-temperature storage [51]. Successful storage of basidiomycete cultures by this method for 30 years was also reported [52]. 

We started using this method for LE-BIN strains in 2000 as a replacement for the outdated, uncomfortable preservation method under Vaseline oil, keeping the vials at room temperature until 2010. Evaluation of strain conditions after 10 years of preservation in water vials at 22–25 °C showed that warm temperatures and some types of screw-cap vials promoted water evaporation from the vials after several years of storage. Control of strain viability showed that only 32% of the checked strains survived after 5–10 years of disk-method preservation. Comparison of our results with data from the literature presented in Table 1 motivated us to change the preservation temperature of the water vials from room to refrigerated temperature (4–6 °C). Our experience showed that this method of preservation is well suited for saprotrophic agaricoid and aphyllophoroid basidiomycetes, whereas gasteromycetes and EMF could not survive long-term preservation under distilled water.

**Figure 3:**
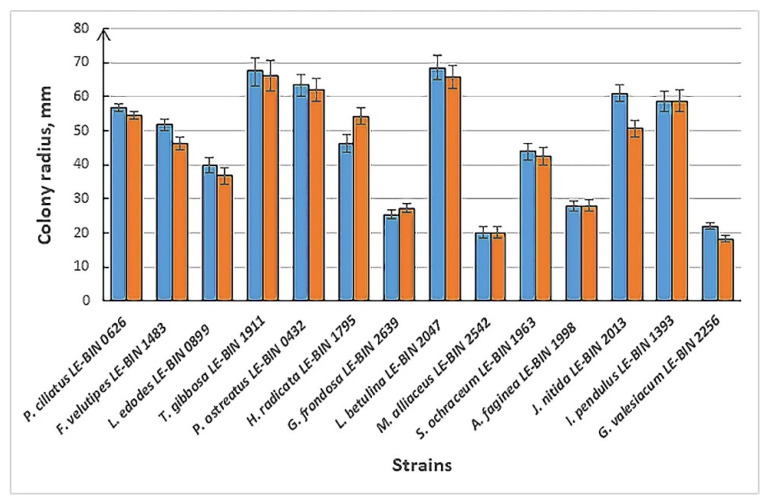
Colony radius of LE-BIN strains on 10th day of growth at 25 °C after 12 months of cryoconservation at −80 °C compared to sub-culture preservation at 5 °C. Blue column—growth after cryo storage; orange column—growth after sub-culture storage.

For the LE-BIN strains, the standard protocol of basidiomycete cryopreservation in screw-cap cryovials under a 10% glycerol–water solution at −80 °C with a freezing rate of 1 °C min^−1^ was used. This modern and reliable method is the most technologically advanced, promising conservation of strains for unlimited time. Although it was shown that even strains of *C. cibarius* can be stored by cryopreservation method [54], strains of EMF and even some saprotroph species need a special approach to achieve success in cryoconservation [55]. This was confirmed after our experiment on the control of viability and growth of some LE-BIN strains after cryoconcervation. Seventeen strains of various species were checked for viability at 1 week and at 1, 3, and 6 months. It was revealed that strains of *Suillus bovinus*, *S. variegatus*, and *Phallus impudicus* lost viability after the first week. Probably, the strains did not survive the initial process of freezing. The rest of the strains were fine and recovered after deep freezing pretty fast. After 12 months of preservation at −80 °C, the strains were tested for viability and growth rate in comparison with the same strains maintained for a year by sub-culture at 5 °C. The results in Figure 3 show that colony radius after 10 days of growth at 25 °C are reliably different in only three pairs of strains—the growth of the *Hymenopellis radicata* strain after cryoconservation was slower, but the growth of the *Flammulina velutipes* and *Junghuhnia nitida* strains was faster in comparison with sub-culture strains. The other strains did not show reliable differences after the two methods.

Recently, we conducted a study to examine the biochemical mechanisms underlying freezing resistance of fungal strains. It was noticed that the strain of *S. lacrymans* LE-BIN 1192 did not always remain viable after 2 weeks of freezing at −20 °C. However, we observed an increase in the freezing resistance of *S. lacrymans* after pre-cultivation of mycelia at elevated (for *S. lacrymans*, it was 25 °C) temperatures [56]. According to the results of lipid and metabolite profiling, triglycerides, sphingolipids, and water-soluble metabolites, such as mannitol, glycerol, sugar alcohols, and some amino- and organic acids, were up-regulated in preheated samples. We hypothesized that they could function as thermoprotective compounds, which might stabilize cellular components, providing a cross-resistance between heat shock and freeze–thaw stress. This finding can be applied in the cryoconservation of freeze-sensitive strains.

### 3.2. Verification Results

#### 3.2.1. Cultural Characteristics

Verification of strains is the next step after obtaining pure cultures. It is an important component of collection work when cultures are used for scientific and, especially, practical purposes. Unfortunately, in practice around the world, especially before the “era of molecular research”, the use of contaminated and misidentified strains was quite common. There may be several reasons for maintaining misidentified cultures in a collection: (1) Incorrect identification of the original basidioma from which the culture was isolated. This often happens when macromycete’s identification is conducted without the participation of taxonomists. (2) The original basidioma belongs to a species complex that has not yet been taxonomically studied. For taxonomic re-identification of the original specimens, it is necessary to preserve them in the herbarium as voucher specimens. (3) Contamination occurred during the culture isolation process. (4) Contamination occurred during storage or sub-culturing of a strain. The strain contamination during storage depends largely on the method of culture preservation in the collections. Currently, the most reliable method for basidiomycetes conservation is cryopreservation in liquid nitrogen. If this method is used correctly, the culture can be stored unchanged for a long time (theoretically indefinitely). However, this method requires expensive equipment and is available only in large collections and resource centers.

Strain verification in the LE-BIN collection is conducted through a complex approach using various methods. Culture characteristics are the basic, obligatory information about strains before their integration into the collection. The culture characteristics of the LE-BIN strains include growth parameters, macro- and micromorphology, and biochemical activity, which help to verify the strains. Often, a cultural study may be enough to reveal contamination and eliminate the strain from the collection. The majority of publications where LE-BIN strains were involved included the description of culture characteristics of studied strains. These include papers on new species from the Russian Far East, *Cruentomycena kedrovayae* R.H. Petersen, Kovalenko & O. Morozova [57]; a bioluminescent fungus, *Neonothopanus nambi* (Speg.) R.H. Petersen & Krisai [58]; a new genus, *Lignomyces*, described in *European Russia* [59]; an interesting tropical basidiomycete fungus, *Rogersiomyces malaysianus* (K. Matsush. & Matsush.) Zmitr. [60]; several recent publications, including a description of a new species, *Marasmiellus boreoorientalis* Kiyashko [61], on biology, taxonomy, and phthalid production of *Lignomyces vetlinianus* (Domański) R.H. Petersen & Zmitr. [15,62]; on rare fungi—a producer of phenol compounds, *Sparassis crispa* (Wulfen) Fr. [17]; on a xylotrophic basidiomycete with phytopathogenic activity, *Sarcodontia setosa* (Pers.) Donk (formerly named *Sarcodontia crocea* (Schwein.) Kotl.), and its biosynthetic potential [63,64]; and on a stenotrophic basidiomycete found in the Republic of Dagestan, *Fomitiporia hippophaeicola* (H. Jahn) Fiasson & Niemelä, which is the causative agent of sea buckthorn (*Hippophaë rhamnoides*) wood rot [65]; and on a xylothrophic fungus, *Fistulina hepatica* LE-BIN 3801 [66].

**Figure 4:**
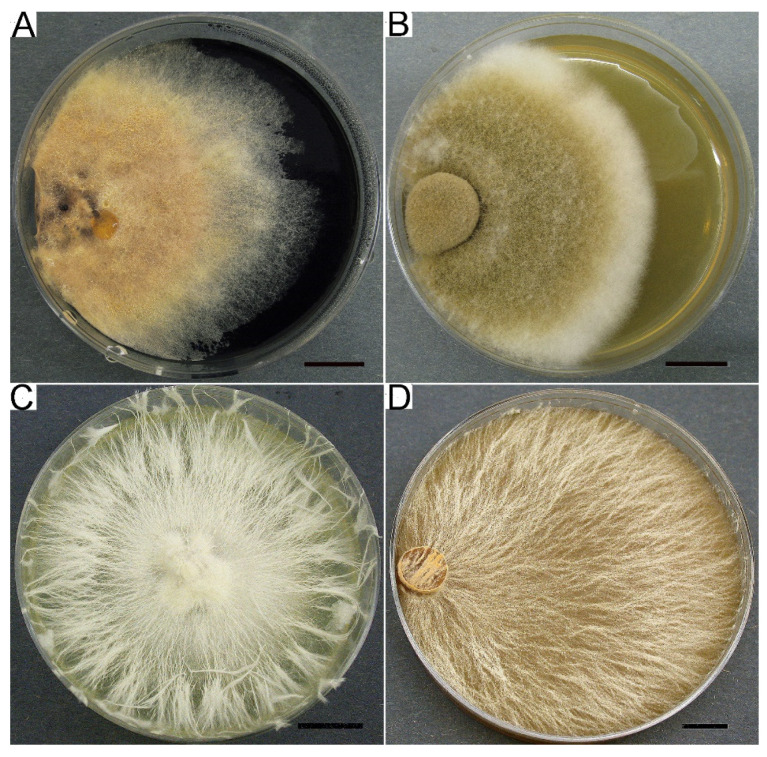
Colony mats of several LE-BIN strains with characteristic macromorphology. (**A**) *Suillus plerans* LE-BIN 2632. (**B**) *Clitocybe martiorum* LE-BIN 2400. (**C**) *Phallus impudicus* LE-BIN 0781. (**D**) *Steccherinum ochraceum* LE-BIN 1963. Scale bars are 10 mm.

**Figure 5:**
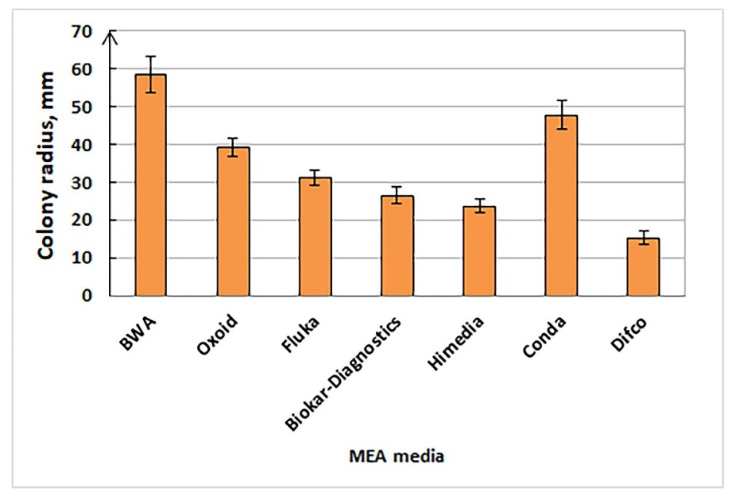
Growth rate of *Pleurotus djamor* LE-BIN 3279 on MEA media of different producers (colony radius (mm) on 10th day of growth).

**Figure 6:**
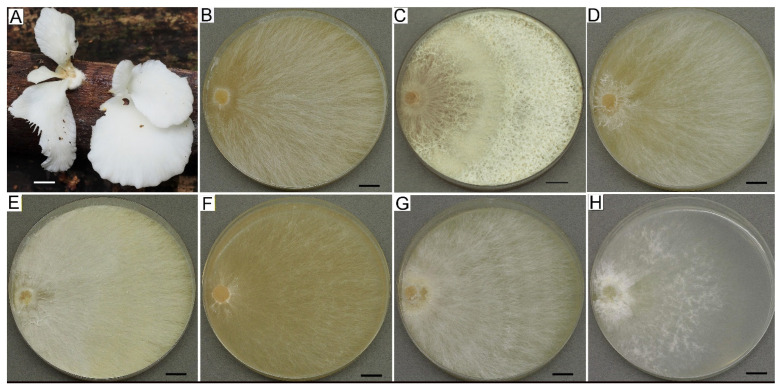
Colony mats of *Pleurotus djamor* LE-BIN 3279 (2 wks of growth) on MEA media of different producers. (**A**) Basidiomata in nature. (**B**) BWA, Russia. (**C**) Oxoid, USA. (**D**) Fluka, Germany. (**E**) Conda, Spain. (**F**) Biokar Diagnostics, France. (**G**) HI-Media, India. (**H**) Difco, USA. Scale bars are 10 mm.

Culture characteristics are the basis of passports of strains, which contain information about collection strains. We are not sure that it is possible to identify macromycete pure cultures by morphological characteristics using only keys and following descriptions, for example, Nobles or Stalpers [5,67], but culture growth, morphology, and biochemical properties can be significantly helpful in strain verification. *Suillus* spp. cultures, for example, are characterized by slow growth, colored fluffy mycelium, darkened media, and specific micromorphology (Figure 4A). Some genera and even species have very specific cultural characteristics and can be easily recognized (e.g., *Clitocybe martiorum* J. Favre, *Terana coerulea*, *Phallus impudicus*, *Pleurotus cystidiosus*, *Steccherinum ochraceum*, *Picnoporus sanguinea*, *Hymenopellis radicata*, *Lentinellus ursinus*). Colony mats of several such examples are shown in Figure 4. It should be noted that culture growth and morphology can be highly dependent on the culture media. Even a single medium produced by different companies caused sufficient differences in growth rate and morphology. This statement can be illustrated by Figure 5 and Figure 6, which show a variety of growth and colony textures of white *Pleurotus djamor*, strain LE-BIN 3279 on MEA.

#### 3.2.2. Fruiting in Culture

**Figure 7:**
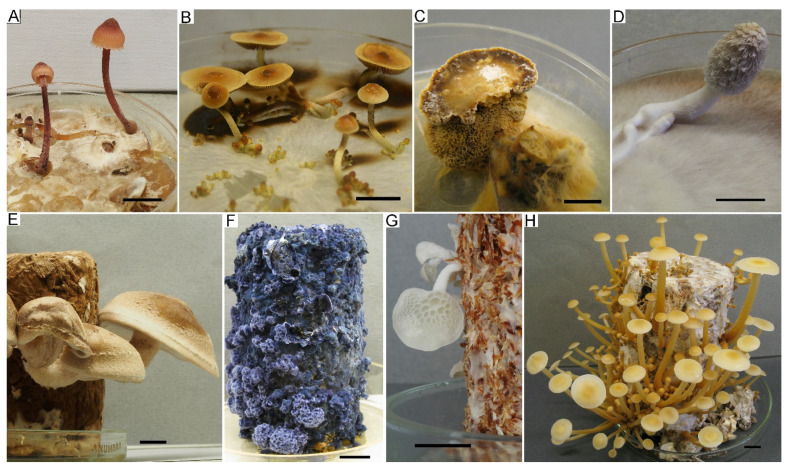
Fruiting in culture on plates (**A**–**D**) and on saw-dust blocks (**E**–**H**) of some LE-BIN strains. (**A**) *Mycena haematopus* LE-BIN 1383. (**B**) *Pholiota highlandensis* LE-BIN 1124. (**C**) *Inonotus obliquus* LE-BIN 0173. (**D**) *Coprinopsis cinerea* LE-BIN 3798. (**E**) *Lentinula edodes* LE-BIN 3339. (**F**) *Terana coerulea* LE-BIN 2820. (**G**) *Favolaschia pustulosa* LE-BIN 3353. (**H**) *Flammulina rossica* LE-BIN 2594. Scale bars are 10 mm.

Fruiting in culture can be a substantial contribution to strain verification. Unfortunately, only a few macromycetes species are capable of fruiting in laboratory conditions. Nevertheless, some saprotrophic and xylotrophic species are able to produce primordia and even mature carpophores on a Petri dish spontaneously if three required mushroom fruiting factors (i.e., temperature, light, and moisture) are present. Often, spontaneous fruiting in plates is limited by forming primordia without their further development. This can be explained by the relatively small amount of nutrients in Petri dishes, which is not enough for developing normal fruiting bodies. In case a strain does not fruit spontaneously, the initiation of the fruiting process can be tried on wooden substrates prepared from a mixture of sawdust and bran. A large number of the LE-BIN strains were cultivated for fruiting, but the results were not always positive. Not everyone studied stains or species has fruited in our conditions. For example, we could not obtain fruiting of gasteromycete fungi, *Neonothopanus*, *Gymnopus*, or strains of some other taxa. Several examples of fruiting in Petri dishes and substrate blocks are presented in Figure 7.

#### 3.2.3. Molecular Analysis

The most reliable method of strain verification is sequencing. The use of PCR analysis in the LE-BIN collection started with sequencing and depositing the NCBI strains that were the subjects of research projects and publications, followed by sequencing of newly arrived strains, and currently, we aim to sequence all strains in the collection. This method provides an opportunity to reveal strains contaminated by other basidiomycete fungi or to clarify the taxonomic affiliation of the strains. Due to this method, tens of strains were eliminated from the collection due to contamination. At the time of writing, over 720 sequences of ITS and LSU regions of LE-BIN strains were deposited in the GenBank NCBI https://www.ncbi.nlm.nih.gov/nuccore (accessed on 5 November 2023). They can be found in the gene bank using a search algorithm by the collection’s acronym LE-BIN. The total number of NCBI sequence depositions related to strains of the LE-BIN collection is 15,738. Besides SSU, 5.8 S, LSU genes, and ITS spacers, in the GenBank are deposited sequences of laccase genes and whole genome sequences of *Steccherinum ochraceum* LE-BIN 3174 [68] and *Lentinula edodes* LE-BIN 0899 [69]. Moreover, the draft genome sequence of *Trametes hirsuta* LE-BIN 072 was published in the *Genome Announcements* [70].

### 3.3. Taxonomical Structure of the LE-BIN Fund

Currently, 3680 strains of macromycetes are maintained in the LE-BIN collection. The majority (appr. 75%) of these strains were identified at the species level and verified by molecular or traditional cultural methods. If sequences of the collection strains provided no homology when blasted in the NCBI, the sequences were compared with ones obtained from their voucher specimens. As a result, the collection’s fund counts 776 species (without forms and varieties). In addition, 21% of the strains were identified up to the generic level. The rest of the strains are still to be determined, which we suppose will increase the taxonomic variety of the collection.

Since the LE-BIN collection was established for the conservation of basidiomycetes and has specialized only on these fungi for many years, they present 98% of the total strain number. A minor amount of strains belong to ascomycetous macrofungi from four classes (Leotiomycetes, Orbiliomycetes, Pezizomycetes, and Sordariomycetes). The most represented are Sordariomycetes (33 strains of 16 species) and Leotiomycetes (32 strains of 17 species). In total, 42 species from Ascomycota are maintained in the collection.

Basidiomycota is represented by 3598 strains from the classes Agaricomycetes, Dacrymycetes, and Tremellomycetes, with the vast majority from the first one. Only one Dacrymycetes strain of *Dacryopinax spathularia* (Schwein.) G. W. Martin (Dacrymycetales, Dacrymycetaceae) and two Tremellomycetes strains of *Phaeotremella foliacea* (Pers.) Wedin, J.C. Zamora et Millanes (Tremellales, Phaeotremellaceae) are preserved in the LE-BIN. The taxonomical structure of Basidiomycota fungi maintained in the collection is illustrated in Table 2, where classes, orders, families, and genera with the number of species and strains (in brackets) are listed.

Agaricomycetes, the largest class of Basidiomycota [19], is the backbone of the collection, presenting in the LE-BIN by 14 orders and 94 families. However, appr. 78% of its strains belong to the two largest orders—Agaricales (1764 strains of appr. 401 species) and Polyporales (1047 strains of appr. 206 species). These orders contain many saprotrophs, especially wood-destroying species, the majority of which can be isolated in culture and preserved ex situ. The other orders containing saprotrophic species are smaller than mentioned above and consequently include noticeably fewer strains and species. For example, 217 strains of over 57 species belong to Hymenochaetales, while only one strain of *Xenasmatella vaga* (Fr.) Stalpers belongs to Xenasmatellales. It should be noted that we also preserve some strains from orders consisting mostly of mycorrhiza-forming uncultured species, i.e., Russulales and Boletales. Russulales is presented by 191 strains of more than 48 species from five non-mycorrhizal families (Auriscalpiaceae, Bondarzewiaceae, Hericiaceae, Peniophoraceae, Stereaceae). The order Boletales, consisting of 30 strains and approximately eight species, includes some members of mycorrhiza-forming families such as Boletaceae (1 strain of *Boletus paluster* Peck) and Suillaceae (3 strains of *Boletinus* and 11 of *Suillus*), as well as the strain of *Amanita phalloides* belonging to Amanitaceae (Agaricales). In total, 16 strains of 12 EMF are currently maintained in the collection.

The two largest orders are also the most represented by families (35 of Agaricales and 25 of Polyporales) and genera (more than 144 and 109, respectively). The largest amount of strains are present in Polyporaceae (395 strains appr. 72 species), Mycenaceae (234 strains, appr. 57 species), Omphalotaceae (198 strains, appr. 37 species), Strophariaceae (196 strains, appr. 43 species), Physalacriaceae (178 strains, appr. 25 species), and Pleurotaceae (165 strains, appr. 18 species).

The rest of the families are distinctly less diverse in both strain and species diversity. It should be noted that there is no strong correlation between strains and species in the collection and the amount of taxa representatives in families and orders because the number of strains and species in the collection may depend on the frequency of species occurrence in nature, the easiness in obtaining and maintaining cultures, the special interests of researchers (i.e., for specific investigation), etc. For example, the small family Pleurotaceae (only appr. 113 species according to He et al. [19]) is represented by a large number of strains. The greatest species diversity is registered for *Mycena* (38 species and 157 strains), *Pholiota* (18 and 92, respectively), *Trametes* (16 and 161), *Pleurotus* (13 and 152), *Marasmius* (11 and 70), *Gymnopus* (11 and 56), and *Collybiopsis* (10 and 46). All these genera, aside from *Pleurotus*, are multi-species, containing many strains being identified only to genus, which means their species variety is really larger. The remaining genera are represented by 9 to 1 strains and do not contain as many unidentified strains or are not multi-species genera.

The largest strain diversity in the collection belongs to *Pleurotus pulmonarius* (Fr.) Quél., with 53 strains from a wide geographic origin. Over 30 strains represent *Pleurotus ostreatus* (Jacq.) P. Kumm. (39 strains) and *Sarcodontia setosa* (Pers.) Donk (32 strains). Two *Pleurotus* species are widely distributed, abundantly fruiting in nature, and easy to culture, while such a number of strains of a rather rare species *S. setosa* can be explained by a special interest in its investigation [63,64]. More than 20 strains of the following species are preserved in the collection: 28 strains of *Lentinula edodes* (Berk.) Pegler, 27 strains of *Ganoderma applanatum* (Pers.) Pat., 27 strains of *Steccherinum ochraceum* (Pers.) Gray, 27 strains of *Trametes versicolor* (L.) Lloyd, 26 strains of *Ganoderma lucidum* (Curtis) P. Karst., 23 strains of *Flammulina velutipes* (Curtis) Singer, 22 strains of *Kuehneromyces mutabilis* (Shaeff.) Singer et A. H. Sm., 22 strains of *Fomitopsis pinicola* (Sw.) P. Karst., 21 strains of *Panellus stipticus* (Bull.) P. Karst., 20 strains of *Bjerkandera adusta* (Willd.) P. Karst., and 20 strains of *Daedaleopsis confragosa* (Bolt.) J. Schröt. These species also either frequently occurred (*B. adusta*, *D. confragosa*, *G. applanatum*, *F. velutipes*, *F. pinicola*, *K. mutabilis*, *P.stipticus*, *T. versicolor*) or are rarer but biotechnologically important (*Ganoderma lucidum*, *L. edodes* and *S. ochraceum*). Approximately half of all species (346) are represented in the collection by only a single strain. But some of them are very rare or recently described, such as *Amanita phalloides* (Vaill. ex Fr.) Link, *Calocybe obscurissima* (A. Pearson) M. M. Moser, *Crepidotus tobolensis* Kapitonov, Biketova et Zmitr., *Gloeoporus hainanensis* Yuan Yuan et Jia J. Chen, *Mycena gombakensis* A. L. C. Chew et Desjardin, *Pseudoarmillariella ectypoides* (Peck) Singer, *Pycnoporellus alboluteus* (Ellis et Everhart) Kotl. et Pouzar, etc., and presumably absent in other fungal collections.

Today, only two *ex-type* strains are maintained in the collection: LE-BIN 4081 *Collybiopsis boreoorientalis* (Kiyashko) Bartrop et Haelewaters [61] and LE-BIN 2000 *Cruentomycena kedrovayae* R.H. Petersen, Kovalenko et O.V. Morozova [57]. Preservation of *ex-type* strains is a very important part of the collection’s activity, and we hope to increase the number of them in the future.

Thus, the considerable strain and species diversity, as well as the presence of some unique strains, make it possible to use the collection in different ways, from systematic and phylogenetic investigations to biotechnological applications.

### 3.4. Biosynthetic and Biotechnological Potential

#### 3.4.1. Oxidase, Cellulase, and Lignin Degradation

Basidial macromycetes are the most important and valuable producers of oxidoreductases, in particular, polyphenol oxidases, especially laccases, peroxidases, tyrosinases, and many other oxidative enzymes that play important roles for human life and industry in many aspects. Interest in oxidases started in the nineteenth century and has continuously increased up to now [71,72,73,74,75,76,77,78]. Strains from the LE-BIN collection have been used to reveal and study oxidoreductases since the late 1970s. Although the current level of oxidase research includes a genomic analysis of a multitude of data available from international databases [79], screening for new prospective producers of oxidative enzymes remains relevant [80,81,82]. Selection of LE-BIN strains for evaluation of oxidative potential was conducted using ecological and taxonomical approaches [83]. It was revealed that typical oxidative enzyme producers were members of the order Polyporales, whose representatives can be divided into four clades: the core polyporoid clade, the phlebioid clade, the antrodia clade, and the residual polyporoid clade [84].

According to published phylogenetic data on Agaricomycetes, in the clusters containing known laccase producers, species with potentially high oxidases were revealed. Taking into account the data obtained, over 550 strains of about 350 aphyllophoroid and agaricoid species maintained in the LE-BIN collection were involved in the screening for oxidases by express methods. The majority of studied strains belonged to xylotrophic white-rot fungi (WRF), but strains of saprotrophs on soil, litter, cones, and some other substrates were also included in experiments. The growth rate of the studied strains was measured and expressed as the number of weeks required for cultures inoculated on the edge to cover 90 mm plates. The results of the screening showed that over 70% of studied basidiomycetes species revealed positive activity of oxidoreductases. High activity was found in over 100 species belonging to various ecological groups, including not only xylotrophic fungi but also litter decomposers and soil saprotrophs. The high activity of white-rot basidiomycete LE-BIN strains from relatively well-studied core polyporoid clade (e.g., genera *Coriolopsis*, *Lentinus*, *Trametes* and *Ganoderma*) was confirmed, and the active strains from the residual polyporoid clade (from genera *Steccherinum*, *Mycorrhaphium*, *Junghuhnia*, *Antrodiella*, *Metuloidea*) was revealed, which is less studied regarding the oxidative potential of its species. Among fast-growing species with high oxidase activity in culture were both aphyllophoroid fungi (i.e., *Trametes hirsuta*, *T. pubescens*, *T. versicolor*, *Abortiporus biennis*, *Datronia mollis*, *Junghuhnia nitida*, *Metuloidea fragrans*, *Steccherinum bourdotii*, *S. ochraceum. Lenzites elegans*, *Peniophora lycii*, *Phellinus gilvus*, *Lentinus substrictus*, and *Trichaptum biforme*) and strains of agaricoid species (i.e., *Agrocybe praecox*, *Mucidula brunneomarginata*, *Leucocybe connata*, *Macrolepiota procera*, *Neonothopanus nambi*, *Pleurotus ostreatus* s.l., and *P. pulmonarius* s.l). The high oxidative potential was revealed in strains of some slowly growing species, such as *Artomyces pyxidatus*, *Hohenbuehelia fluxilis*, *Strobilurus tenacellus*, *Mycetinis scorodonius*, *Atheniella flavoalba*, other mycenoid species, and some *Gymnopus* and marasmioid species. 

As a result of the express analysis, the most active strains were cultivated in submerged conditions and studied for laccase production, e.g., *Panus rudis* Fr. LE-BIN 1566, *Trametes hirsuta* (Wulfen) Lloyd LE-BIN 072, *T. gibbosa* (Pers.) Fr. LE-BIN 1911, *Coriolopsis caperata* (Berk.) Murrill (*Cerrena caperata* (Berk.) Zmitr.) LE-BIN 0677, *Lenzites betulinus* (L.) Fr. LE-BIN 2047, and some other strains from the core polyporoid clade [85,86]. Of particular interest was the cultivation and study representatives from the residual polyporoid clade, e.g., strains from the family Steccherinaceae—*Steccherinum ochraceum* (Pers. ex J.F. Gmel.) Gray LE-BIN 1833, *S. bourdotii* Saliba & A. David LE-BIN 2738, and *Junghuhnia nitida* (Pers.) Ryvarden LE-BIN 2013. For the first time, laccases were isolated and characterized from these species [85,87]. Screening for strains with high laccase activity revealed species from Polyporaceae and Steccherinaceae that have a great biotechnological potential. One of the practical results of this work was the strain *Steccherinum ochraceum* LE-BIN 1833, which produced thermostable and acidophilic laccases, which allows their use in biotechnological processes at high temperatures and low pH values [88]. The strain variety of *S. ochraceum*, *S. bourdotii*, and other *Steccherinum* species in the LE-BIN collection (39 strains of five species in total) opens a great potential for further investigation of these fungi as oxidoreductase producers.

Xylotrophic basidiomycetes play a central role in wood decomposition and are active participants in the global carbon cycle because they possess unique extracellular enzymes to destroy hard-to-degrade lignocellulose [89]. The process of decomposition of resistant plant polymers by basidiomycetes is carried out by two types of extracellular multienzyme complexes: ligninolytic, involving oxidative enzymes that can degrade lignin, and hydrolytic, consisting of cellulolytic and hemicellulolytic enzymes responsible for the depolymerization of polysaccharides [90,91,92]. Due to the enzymatic mechanisms of detoxification of not only lignocellulose degradation products but also of various xenobiotics, these fungi have a wide range of applications in various fields of biotechnology [93].

To select the most prospective strains for utilization of lignin-containing waste, over 120 strains of 60 species of xylotrophic aphyllophoroid fungi were screened for lignolytic and cellulolytic enzymes. Fungi growing on live and dead wood were collected and isolated in culture during the field trips in 2015–2020. The distribution of taxa, including active producers of enzymes of the lignocellulolytic complex in the modern system of fungi, was revealed. It was shown that fungal species with high lignin and/or cellulolytic potential could be divided into two ecological and trophic groups inhabiting wood of different decomposition degrees in natural conditions. Metadata are presented in the Supplementary Materials (Table S1). The first group included obligate and facultative pathogens from the genera *Hymenochaetaceae* (*Inonotus hispidus*), *Meruliaceae* (*Sarcodontia setosa*), and *Peniophoraceae* (*Peniophora cinerea*, *P. incarnata*, *P. quercina*) and primary xylosaprotrophs from the genera *Gloeophyllaceae* (*Gloeophyllum trabeum*) and *Polyporaceae* (*Funalia trogii*, *Trametes quercina*), which initiate the process of the decomposition of wood of fallen or dead trees. The second group consisted of members of the genera *Irpicaceae* (*Irpex lacteus*, *Raduliporus aneirinus*), *Polyporaceae* (*Lentinus arcularius*), and *Steccherinaceae* (*Junghuhnia nitida*, *Metuloidea fragrans*, *Steccherinum bourdotii*, *S. ochraceum*), which are secondary xylosaprotrophs that prefer small wood fragments (branches, thin trunks, etc.) or decayed wood [94,95]. Information on the genetic and enzymatic mechanisms managing the adaptation of *S. ochraceum* LE-BIN 3174 to late stages of wood decomposition was obtained [96]. The study of *Steccherinum* strains was continued by evaluation of the oxidative enzyme activity and biodegradation potential of seventeen LE-BIN strains of *S. ochraceum* during their cultivation on different types of wood [97].

The test with the polyphenolic dye azure B detected fungi with high degradation potential, prospective for applications in bioconversion and bioremediation, and revealed strains of both white- and brown-rot fungi from the families *Irpicaceae* (*Gloeoporus dichrous*), *Meruliaceae* (*Phlebia rufa*), *Polyporaceae* (*Trametes gibbosa*, *T. pubescens*), *Fomitopsidaceae* (*Fomitopsis betulina*, *Pycnoporellus fulgens*), *Hymenochaetaceae* (*Inocutis rheades*, *Phellinus laevigatus*), and *Hericiaceae* (*Laxitextum bicolor*) [94]. 

Of great importance is the study of indoor fungi strains regarding the problem and economic significance of indoor wood-rot fungi causing enormous damage to industrial wood and within wooden buildings [98]. Despite a rather long study of house fungi, revealing their new biological features and the ability to adapt to the anthropogenic environment and cause intensive destruction of commercial wood in various environmental conditions are still of considerable interest. It is obvious that studies of the biosynthetic properties of strains of key indoor fungi species can contribute to understanding their adaptive potential and help to prevent the destructive effect of these fungi on commercial wood and wooden buildings. The LE-BIN strains of key species of indoor macrofungi (i.e., *Amyloporia xantha*, *Coniophora puteana*, *Gloeophyllum sepiarium*, *Neolentinus lepideus*, *Serpula lacrymans*, and *S. himantioides*) were studied for cellulolytic, proteolytic, and acidifying activity. It was shown that the strains of *C. puteana*, *S. lacrymans*, and *A. xantha* were characterized by the greatest cellulolytic and acidifying activities [99].

#### 3.4.2. Proteinases

Strains of various taxonomical groups from the LE-BIN collection have been studied for proteolytic activity, with an emphasis on thrombolytic, fibrinolytic, and gelatinase activities since the 1970s. Over 700 strains were studied using the application method on fibrinolytic activity. The most active strains were found in Polyporaceae, Schizophyllaceae, Pleurotaceae, Coprinaceae, and Physalacriaceae families. All active species of apphyllophoroid fungi were representatives of WRF; most active agaricoid strains were saprotrophs on wooden or rich organic substrates [100]. Strains with high activity in terms of proteolytic enzymes were cultivated on liquid media in static and submerged conditions to evaluate their production of thrombolytic and fibrinolytic exoenzymes. The most studied and prospective strains were the *Flammulina velutipes*, *Cerrena unicolor*, *Coprinus domesticus* (=*Coprinellus domesticus*), and *Coprinus cinereus* (=*Coprinopsis cinerea*) strains [101,102,103]. Thrombo- and fibrinolytic enzymes were isolated and studied in detail. It was shown that basidial fungi synthesized four types of proteinases—aspartyl, serine, cysteine, and metalloproteinase—and their combination varied in different taxa and correlated with fungal trophic groups. The main contribution to the fibrinolytic effect was made by neutral metalloproteinases [104,105,106].

LE-BIN strains of indoor fungi were evaluated for proteolytic enzymes using gelatin film as substrate. It was shown that the gelatinase activity of strains of all studied species (i.e., *Coniophora puteana*, *Gloeophyllum sepiarium*, *Serpula lacrymans*, *S. himantioides*, and *Neolentinus lepideus*) was positive, excluding two strains of *N. lepideus*, LE-BIN 0963 and LE-BIN 2278, where no lysis zones on the gelatin film were observed. The strains *C. puteana* LE-BIN 1370 and *G. sepiarium* LE-BIN 2059 were characterized by relatively high gelatinase activity. The diameters of their lysis zones on the gelatin substrate were 28.0 ± 0.3 and 25.9 ± 0.1 mm, respectively [99].

#### 3.4.3. Lipids

Edible fungi are considered healthy foods because of their good balance of carbohydrates and proteins and low fat concentrations. Although basidiomycetous fungi are rarely used as oil producers, they contain essential fatty acids such as linoleic and linolenic acids in their lipid profiles, usually as the major constituents [107,108]. Omega-3 and omega-6 polyunsaturated fatty acids (PUFAs) have multiple biological roles in human organisms, such as influencing the inflammatory cascade, reducing blood cholesterol and oxidative stress, and providing neuroprotection and cardiovascular protection [109,110]. The most cultivated and commercialized mushrooms are from *Agaricus*, *Lentinula*, and *Pleurotus* genera; however, different edible fungi show significant differences in lipid composition [111]. Recently, the LE-BIN collection strains were screened for producers of essential lipids [14]. Lipidomic profiling of 38 fungal strains belonging to Agaricales (12 strains), Polyporales (17), Russulales (5), Gleophyllales (2), Cantharellales (1), Auriculariales (1), and Phallales (1) from the LE-BIN collection demonstrated that most of the strains are sources of phospholipids rich in PUFAs, namely linoleic C18:2 and linolenic C18:3 acids. Phospholipids of studied strains consisted of 70–80% linoleic acid. Among edible fungi, the strain of *Flammulina velutipes* LE-BIN 1483 was demonstrated to have the maximum amount of linolenic acid (10% of 18:2/18:3 molecular species of phosphatidylcholines), with a total lipid content of 9–11% of dry weight.

Surprisingly, five strains, i.e., *Sparassis crispa* LE-BIN 2902, *Tyromyces lacteus* LE-BIN 3990, *Dentipellis fragilis* LE-BIN 3869, *Irpex lacteus* LE-BIN 4341, and *Laetiporus sulphureus* LE-BIN 3867, synthesized a high proportion of monounsaturated (MUFA) polar lipids, esterified predominantly by oleic C18:1 fatty acid. MUFA is the best choice for biodiesel production and among fungi usually produced by yeasts [112].

One of the most rare and interesting groups of lipids are acetylenic fatty acids containing one or more carbon–carbon triple bonds. Several acetylenic fatty acids (e.g., tariric, pyrulic, crepenic, stearolic, exocarpic, dihydrooropheic acids) found in the seeds of rare plants may potentially have antioxidant and other biological activities [113]. To our knowledge, currently, the absence of commercial oils containing acetylenic acids from natural plant material may be due to economic reasons. Besides plants, fungi also have the ability to produce acetylenic acids, but all studies in this regard were carried out on natural fruiting bodies but not on pure cultures. In the LE-BIN collection, we discovered a new oleogenic strain. In the experiments conducted, a tropical strain, *Rogersiomyces malaysianus* LE-BIN 3507, contained triglycerides of 40% esterified with acetylene dehydrocrepenynic fatty acid. The culture demonstrated active growth and accumulated up to 25% of triglycerides from the dry mass of the mycelium (without technological optimization). The rare basidiomycete species [60] has not been studied for its lipid content before and has been declared to be oleogenic for the first time. It may be of commercial interest for further investigation as a potential producer in biotechnology.

#### 3.4.4. Secondary Metabolites

It is well known that fungi produce thousands of different secondary metabolites—phenolic compounds, alkaloids, terpenoids, etc. Studies of unusual compounds were carried out on *Lignomyces vetlinianus* LE-BIN strains producing visible crystal-like conglomerates in Petri dishes. These substances were picked up from the plates and identified by gas chromatography mass-spectrometry (GS-MC) as clusters of 4,6-dimethoxy-phthalide and 4, 6-dimetoxy-1(3H)-isobenzofuranone, which were also found in the mycelium. The molecular structure of the substance was confirmed by nuclear magnetic resonance (NMR) analysis [15]. These substances are considered useful and are known to be contained in some plants, for example, celery (*Apium graveolens*) and a number of microorganisms, but among Agaricomycetes, they are very rare [114].

Crystals produced by *Sparassis crispa* strains were identified using GS-MC and NMR methods as sparassol (methyl 2-hydroxy-4-methoxy-6-methyl benzoate) and its derivatives (methyl ester of sparassol and methyl orsellinate). The strain *S. crispa* LE-BIN 2902 formed crystals of various sizes and shapes most intensively [17]. Sparassol is known to have antibiotic activity, and the strain of rare and medicinal mushrooms actively synthesizing sparassol may be of interest for further study.

A comprehensive study in collaboration with Saint Petersburg State Chemical Pharmaceutical University and Saint Petersburg Pasteur Institute (Russia) included investigation of secondary metabolites produced by *Sarcodontia setosa* and *Fistulina hepatica* using spectroscopic methods (UV, NMR, and high resolution-electrospray ionisation-mass-spectrometry (HR-ESIMS)). Five compounds from a cultural liquid after cultivation of *S. setosa* LE-BIN 4350 were isolated, and their chemical structures were determined as two new derivatives of sarcodontic acid—setosic acid and 7,8-dehydrohomosarcodontic acid—and as three previously known benzoquinone pigments. Screening tests of the antibacterial activity of these compounds against so-called ESKAPE (*Enterococcus faecium*, *Staphylococcus aureus*, *Klebsiella pneumoniae*, *Acinetobacter baumannii*, *Pseudomonas aeruginosa*, and *Enterobacter* species) bacterial strains were carried out in vitro, and the biosynthetic relationships of the obtained compounds were discussed. *K*. *pneumoniae*, S. *aureus*, and *A. baumannii* were found to be most sensitive to compounds from *S. setosa* [115]. The investigation of the polypore fungus *F. hepatica* LE-BIN 3801 revealed four new polyacetylenic fatty acid derivatives—isocinnatriacetin, isocinnatriacetin A, cinnatriacetin C, and ethylcinnatriacetin A. Screening of the antibacterial activity of the produced compounds against ESKAPE bacterial strains in vitro with the determination of inhibition zones showed that the compounds from *F. hepatica* demonstrated the highest efficacy against *E*. *faecium*, S. *aureus*, and *K*. *pneumoniae* [66].

One of the important groups of terpenoids is triterpenoids—polycyclic compounds derived from the straight-chain hydrocarbon squalene. Fungal triterpenoids were reported to possess diverse biological activities such as anti-cancer, anti-inflammatory, immunomodulatory, and anti-obesity activities [116]. Lanostane-type triterpenoid acids—a group of tetracyclic triterpenoids derived from lanosterol—are bioactive components of such well-known medicinal fungi as *Laetiporus sulphureus* [117] and *Wolfiporia cocos* [118]. Pharmacological investigations demonstrated that eburicoic and dehydroeburicoic acids isolated from *Antrodia camphorata* possessed analgesic, anti-inflammatory, and hepatoprotective effects [119,120]. In another study on *A. camphorate*, it was shown that dehydroeburicoic acid prevents the diabetic and dyslipidemic state [121]. Kim et al. [122] demonstrated that trametenolic acid from *Inonotus obliquus* has modest cytotoxic effects against L1210 cells. Dehydrotrametenolic acid from *Wolfiporia cocos* selectively inhibited the growth of H-ras-transformed rat2 cells and induced apoptosis through the caspase-3 pathway [123].

In a recent study, LE-BIN strains of 97 basidiomycete species were screened for triterpenoid acids. It was found in 15 strains producing four lanosterol-type triterpene carboxylic acids, namely trametenolic acid (3ß-Hydroxy-lanosta-8,24-dien-21-oic acid), dehydrotrametenolic acid (3ß-Hydroxy-lanosta-7,9(11),24-trien-21-oic acid), eburicoic acid (3ß-hydroxy-24-methylene-8-lanostene-21-oic acid), and dehydroeburicoic acid (3ß-Hydroxy-24-methylenelanosta-7,9(11)-dien-21-oic acid) (Table 3). Out of the four compounds, eburicoic acid prevailed in content, whereas trametenolic acid was found to dominate only in the *Neolentinus lepideus* LE-BIN 4332 strain [18].

Strains of macromycetes that can be isolated in culture, characterized by fast growth and intensive production of similar secondary metabolites, are of interest for biotechnological research.

### 3.5. Medical and Veterinary Aspects of LE-BIN Strains Application

As a result of screenings of the LE-BIN strains for targeted activity or compounds, the most active and well-growing strains were considered producers for medicine or biotechnology. These strains were studied in submerged conditions with optimized medium and cultivation conditions for maximum production of targeted compounds.

In the last third of the 20th century, the trend of searching and studying enzymes of fibrino- and thrombolytic activity was actively developing. The narrow range of thrombolytic drugs of microbial origin and the presence of serious side effects in them, primarily due to the high level of general, nonspecific proteolysis and sporulation of the producer, hindered their use in medical practice. Active strains of *Flammulina velutipes*, *Coprinopsis cinerea*, and *Cerrena unicolor* were used as producers for fibrinolytic drugs, which passed successful tests on animals in vivo [106]. Several authors’ certificates were received in Russia for the development of fibrino- and thrombolytic drugs based on the LE-BIN strains, including the development of a method for producing thrombus-resistant polymer materials needed for the production of artificial organs for medicine—vascular prostheses, heart valves, catheters, etc. [124,125,126]. The search and study of new producers of thrombolytic enzymes remains an urgent problem in practical biomedicine today, and it would be useful in modern research to rely on the already accumulated experience in this area [127].

The antiviral activity of macromycetes has been attracting much attention since, in 1979, Takehara reported about the antiviral activity of virus-like particles extracted from *Lentinula edodes* [128]. Later antiviral activity against various viruses was found in many basidiomycota, both in agarics and in polypore fungi [129]. Eleven LE-BIN strains of polyporoid macromycetes were studied for antiviral activity against flu viruses (the avian and the human influenza viruses). Various levels of antivirus activity were detected for the majority of studied strains, while the most active were strains of *Daedaleopsis confragosa*, *Datronia mollis*, *Ischnoderma benzoinum*, *Ganoderma valesiacum*, *Trametes gibbosa*, *T. versicolor*, *Laricifomes officinalis*, and *Lenzites betulinus*. The best activity against human influenza was registered for *Daedaleopsis confragosa* LE-BIN 2266 and *Ischnoderma bensoinum* LE-BIN 2264. These strains were the most promising for further investigation of antiviral properties [130]. Currently, the interest in medicinal mushrooms and their antiviral properties is growing due to the emergence of new threats like COVID-19, SARS, MERS, etc. Fungi, with their terpenoids, lectins, glycoproteins, lentinan, galactomannan, and polysaccharides, are considered promising producers of prophylactic or therapeutic agents against these viruses [131,132,133].

The possibility of using fungal strains in veterinary medicine was demonstrated in the example of a medicinal drug based on sterile culture filtrate of *Trametes pubescens* strain LE-BIN 0663. The drug had high therapeutic efficacy in the treatment of gastrointestinal diseases in calves. A Russian patent was received for this scientific research [134].

Over the long history of the LE-BIN collection, strains of macromycetes have been widely and successfully used in various biotechnological research. A total of 27 authors’ certificates and patents have been registered for various scientific inventions with applications of the LE-BIN strains, and about 700 strains have been the subject of research in scientific publications. In recent decades, due to the development and availability of molecular research methods, it has become possible to reliably identify strains, which minimizes the cases of biotechnological application of incorrectly identified or contaminated producers. Biotechnological application of fungal strains isolated and maintained by specialists and preserved in culture collections allows for solving many problems related to their identification, stability, and productivity.

### 3.6. Information on the LE-BIN Fungi (Databases and Catalogs)

The first list of the LE-BIN strains was published in 1980, and it included over 500 strains from 194 genera of aphyllophoroid, agaricoid, boletoid, and gasteroid fungi [135]. The first issue of the collection catalog was published in 1992 and included 470 strains of 300 species from 126 Agaricomycetes genera [136]. The second issue of the LE-BIN catalog was published in 2007 with information about 1463 strains of over 500 species from 200 genera [137]. The up-to-date information about the LE-BIN collection and a partial catalog of the LE-BIN strains are available on several websites. The home page of the LE-BIN is on the website of the Komarov Botanical Institute of the Russian Academy of Sciences (www.binran.ru (accessed on 5 November 2023)). LE-BIN is a member of WFCC (LE-BIN 1015) and a part of the WDCM project Global Catalogue of Microorganisms (https://gcm.wdcm.org (accessed on 5 November 2023)). In 2022, it became a partner of the network of Russian microbial collections on the basis of the All-Russian Collection of Microorganisms (VKM) (https://vkm.ru/Collections.htm (accessed on 5 November 2023)). Available fungi can be found following the above links using the collection’s acronym. Detailed information on purchase and exchange procedures, as well as prices of strains, can be received on request.

## Figures and Tables

**Table 1:** Viability of strains after preservation under distilled water by disk method.

Number of Strains	Survival (%)	Storage t (°C)	Storage Time (Years)	Reference
151	94	5	7	[50]
34	88	5	20	[51]
393	26	20	10	[53]
159	32	22–25	5–10	LE-BIN strains

**Table 2:** Classes, orders, families, and genera of Basidiomycota in the LE-BIN. The number of species and strains (in brackets) is provided after the genus name.

Class	Order	Family	Genus
Agaricomycetes			
	Agaricales	Agaricaceae	*Agaricus* 8 (36), *Chlorophyllum* 2 (3), *Coprinus* 2 (5), *Disciseda* 1 (1), *Lepiota* 1 (2), *Leucoagaricus* 3 (6), *Macrolepiota* 2 (8), *Micropsalliota* 1 (1), *Montagnea* 1 (3), *Mycenastrum* 1 (3)
		Amanitaceae	*Amanita* 1 (1)
		Battarreaceae	*Tulostoma* 3 (5)
		Bolbitiaceae	*Bolbitius* 1 (1), *Conocybe* 1 (3)
		Callistosporiaceae	*Callistosporium* 1 (2)
		Clitocybaceae	*Clitocybe* 8 (29), *Lepista* 1 (2)
		Crepidotaceae	*Crepidotus* 5 (15), *Simocybe* 1 (1)
		Cyphellaceae	*Atheniella* 2 (5), *Baeospora* 1 (2), *Chondrostereum* 1 (6), *Gloeostereum* 1 (2), *Granulobasidium* 1 (1), *Hemimycena* 1 (4), *Henningsomyces* 1 (1), *Mycopan* 1 (1)
		Entolomataceae	*Clitopilopsis* 1 (1), *Clitopilus* 3 (5), *Entoloma* 1 (2)
		Fayodiaceae	*Conchomyces* 1 (2)
		Galeropsidaceae	*Panaeolina* 1 (1), *Panaeolus* 5 (16)
		Hygrophoraceae	*Arrhenia* 2 (4), *Lignomphalia* 1 (1), *Pseudoarmillariella* 1 (1)
		Hymenogastraceae	*Flammula* 2 (8), *Galerina* 9 (27), *Gymnopilus* 8 (37), *Hemistropharia* 1 (1), *Psilocybe* 5 (24)
		Lycoperdaceae	*Apioperdon* 1 (13), *Bovista* 4 (15), *Bovistella* 1 (4), *Calvatia* 3 (3), *Lycoperdon* 6 (38)
		Lyophyllaceae	*Asterophora* 1 (1), *Calocybe* 2 (3), *Hypsizygus* 3 (19), *Lyophyllum* 5 (10), *Ossicaulis* 2 (13), *Tephrocybe* 1 (2), *Termitomyces* 1 (1)
		Marasmiaceae	*Campanella* 1 (2), *Chaetocalathus* 1 (1), *Crinipellis* 1 (2), *Marasmius* 11 (70), *Paramarasmius* 1 (1), *Tetrapyrgos* 1 (4)
		Mycenaceae	*Cruentomycena* 1 (2), *Dictyopanus* 1 (5), *Favolaschia* 3 (9), *Filoboletus* 1 (3), *Heimiomyces* 1 (1), *Mycena* 38 (157), *Panellus* 5 (29), *Roridomyces* 1 (1), *Xeromphalina* 6 (27)
		Nidulariaceae	*Crucibulum* 1 (8), *Cyathus* 6 (31), *Nidula* 1 (1), *Nidularia* 1 (5)
		Omphalinaceae	*Infundibulicybe* 2 (9), *Omphalina* 1 (1)
		Omphalotaceae	*Anthracophyllum* 1 (1), *Collybiopsis* 10 (46), *Connopus* 1 (1), *Gymnopus* 11 (56), *Lentinula* 2 (29), *Marasmiellus* 2 (5), *Mycetinis* 3 (22), *Neonothopanus* 1 (9), *Omphalotus* 2 (10), *Paragymnopus* 1 (4), *Pseudomarasmius* 1 (1), *Rhodocollybia* 2 (14)
		Phyllotopsidaceae	*Phyllotopsis* 1 (7)
		Physalacriaceae	*Armillaria* 5 (52), *Cylindrobasidium* 1 (2), *Flammulina* 9 (57), *Hymenopellis* 2 (19), *Mucidula* 2 (30), *Oudemansiella* 1 (3), *Rhizomarasmius* 2 (3), *Rhodotus* 1 (5), *Xerula* 2 (7)
		Pleurotaceae	*Hohenbuehelia* 3 (5), *Lignomyces* 1 (7), *Pleurotus* 13 (152), *Resupinatus* 1 (1)
		Pluteaceae	*Volvariella* 1 (1)
		Porotheleaceae	*Clitocybula* 2 (7), *Delicatula* 1 (3), *Gerronema* 1 (3), *Hydropus* 3 (12), *Megacollybia* 3 (20), *Trogia* 1 (4)
		Psathyrellaceae	*Candolleomyces* 2 (10), *Coprinellus* 7 (33), *Coprinopsis* 9 (21), *Cystoagaricus* 1 (1), *Parasola* 1 (1), *Psathyrella* 5 (27)
		Pseudoclitocybaceae	*Clitopaxillus* 1 (1)
		Pterulaceae	*Pterulicium* 1 (1)
		Radulomycetaceae	*Radulomyces* 3 (6)
		Sarcomyxaceae	*Sarcomyxa* 1 (4)
		Schizophyllaceae	*Schizophyllum* 2 (24)
		Squamanitaceae	*Cystoderma* 1 (5), *Phaeolepiota* 1 (2)
		Strophariaceae	*Agrocybe* 6 (18), *Deconica* 3 (7), *Hypholoma* 6 (31), *Kuehneromyces* 2 (24), *Leratiomyces* 3 (5), *Melanotus* 1 (4), *Pholiota* 18 (92), *Protostropharia* 1 (6), *Stropharia* 3 (9)
		Tricholomataceae	*Leucopaxillus* 2 (5)
		Tubariaceae	*Cyclocybe* 2 (3), *Tubaria* 1 (4)
		Incertae sedis	*Aspropaxillus* 1 (1), *Collybia* 3 (16), *Cynema* 1 (2), *Cystodermella* 3 (6), *Fistulina* 1 (7), *Leucocortinarius* 1 (1), *Leucocybe* 2 (3), *Meottomyces* 1 (1), *Notholepista* 1 (1), *Paralepista* 1 (1), *Rhizocybe* 1 (1), *Ripartites* 1 (1)
		Unspecified	“*Coprinus*” (12)
	Amylocorticiales	Amylocorticiaceae	*Ceraceomyces* 1 (1), *Irpicodon* 1 (2), *Plicaturopsis* 1 (2)
	Auriculariales	Auriculariaceae	*Alloexidiopsis* 1 (1), *Auricularia* 6 (21), *Elmerina* 2 (4), 1 (1)
		Incertae sedis	*Guepinia* 1 (1), *Protomerulius* 1 (1), *Pseudohydnum* 1 (2)
	Boletales	Boletaceae	*Boletus* 1 (1)
		Coniophoraceae	*Coniophora* 1 (7),
		Hygrophoropsidaceae	*Hygrophoropsis* 1 (2)
		Serpulaceae	*Serpula* 2 (4)
		Suillaceae	*Boletinus* 1 (3), *Suillus* 9 (11)
		Tapinellaceae	*Pseudomerulius* 1 (1), *Tapinella* 1 (1)
	Cantharellales	Hydnaceae	*Rogersiomyces* 1 (1), *Sistotrema* 1 (6)
	Corticiales	Punctulariaceae	*Punctularia* 1 (3)
		Vuilleminiaceae	*Vuilleminia* 1 (3)
	Geastrales	Geastraceae	*Geastrum* 1 (3), *Sphaerobolus* 2 (4)
	Gloeophyllales	Gloeophyllaceae	*Gloeophyllum* 5 (24), *Neolentinus* 3 (21)
	Gomphales	Clavariadelphaceae	*Clavariadelphus* 1 (1)
		Gomphaceae	*Ramaria* 2 (4)
		Lentariaceae	*Hydnocristella* 1 (1), *Lentaria* 1 (2)
	Hymenochaetales	Hirschioporaceae	*Hirschioporus* 2 (9), *Pallidohirschioporus* 1 (16)
		Hymenochaetaceae	*Fomitiporella* 1 (1), *Fomitiporia* 2 (7), *Fulvifomes* 1 (2), *Fuscoporia* 3 (5), *Hydnoporia* 1 (1), *Hymenochaete* 5 (7), *Hymenochaetopsis* 1 (2), *Inocutis* 3 (8), *Inonotus* 4 (24), *Phellinopsis* 1 (2), *Phellinus* 8 (70), *Phylloporia* 1 (2), *Porodaedalea* 4 (8), *Pyrrhoderma* 1 (2), *Sanghuangporus* 1 5), *Tropicoporus* 1 (2), *Xanthoporia* 1 (3)
		Hyphodontiaceae	*Hyphodontia* 3 (8)
		Oxyporaceae	*Oxyporus* 3 (10)
		Rickenellaceae	*Peniophorella* 1 (1)
		Schizoporaceae	*Schizopora* 1 (3), *Xylodon* 6 (13)
		Trichaptaceae	*Pseudotrichaptum* 1 (2)
		Unspecified	“*Trichaptum*” (4)
	Phallales	Phallaceae	*Clathrus* 1 (1), *Mutinus* 2 (2), *Phallus* 4 (20)
	Polyporales	Adustoporiaceae	*Adustoporia* 1 (1), *Amyloporia* 1 (6), *Rhodonia* 1 (4)
		Auriporiaceae	*Auriporia* 1 (1)
		Cerrenaceae	*Cerrena* 3 (22)
		Climacocystaceae	*Climacocystis* 1 (2)
		Fibroporiaceae	*Fibroporia* 1 (1)
		Fomitopsidaceae	*Antrodia* 4 (10), *Daedalea* 2 (7), *Fomitopsis* 3 (41), *Neoantrodia* 2 (2), *Ranadivia* 1 (1), *Rhodofomes* 2 (16), *Rhodofomitopsis* 1 (3)
		Ganodermataceae	*Ganoderma* 7 (77), *Sanguinoderma* 1 (1)
		Grifolaceae	*Grifola* 1 (7)
		Hyphodermataceae	*Hyphoderma* 3 (7), *Mutatoderma* 1 (5)
		Incrustoporiaceae	*Skeletocutis* 3 (4), *Tyromyces* 3 (7)
		Irpicaceae	*Byssomerulius* 1 (6), *Ceriporia* 2 (9), *Efibula* 1 (4), *Flavodon* 1 (3), *Gloeoporus* 1 (1), *Irpex* 3 (21), *Leptoporus* 1 (4), *Resiniporus* 1 (1), *Trametopsis* 1 (5), *Vitreoporus* 1 (8)
		Ischnodermataceae	*Ischnoderma* 1 (10)
		Laetiporaceae	*Laetiporus* 3 (20)
		Laricifomitaceae	*Laricifomes* 1 (7)
		Meripilaceae	*Meripilus* 1 (4), *Physisporinus* 1 (3), *Rigidoporus* 3 (5)
		Meruliaceae	*Aurantiporus* 1 (3), *Ceriporiopsis* 1 (2), *Climacodon* 2 (10), *Crustodontia* 1 (1), *Hermanssonia* 1 (2), *Hydnophlebia* 1 (4), *Lilaceophlebia* 1 (1), *Merulius* 1 (2), *Mycoacia* 2 (4), *Pappia* 1 (5), *Phlebia* 4 (31), *Phlebiodontia* 1 (1), *Phlebiopsis* 2 (2), *Sarcodontia* 1 (32)
		Panaceae	*Cymatoderma* 2 (2), *Panus* 3 (23)
		Phaeolaceae	*Phaeolus* 1 (9)
		Phanerochaetaceae	*Bjerkandera* 2 (26), *Hapalopilus* 2 (6), *Phanerochaete* 2 (15), *Porostereum* 1 (5), *Rhizochaete* 1 (3), *Terana* 1 (1)
		Podoscyphaceae	*Abortiporus* 1 (1), *Podoscypha* 1 (1)
		Polyporaceae	*Abundisporus* 1 (1), *Cellulariella* 2 (9), *Cerioporus* 4 (14), *Cubamyces* 3 (8), *Daedaleopsis* 4 38), *Earliella* 1 (1), *Echinochaete* 1 (1), *Favolus* 3 (8), *Fomes* 2 (13), *Funalia* 2 (2), *Grammothele* 1 (1), *Haploporus* 1 (1), *Hexagonia* 1 (1), *Lentinus* 8 (47), *Lenzites* 1 (9), *Lopharia* 1 (1), *Microporellus* 1 (1), *Microporus* 2 (21), *Neofavolus* 2 (8), *Neofomitella* 1 (1), *Perenniporia* 1 (2), *Picipes* 4 (15), *Podofomes* 2 (4), *Polyporus* 3 (20), *Poronidulus* 1 (3), *Pseudofavolus* 1 (2), *Pyrofomes* 1 (1), *Szczepkamyces* 1 (1), *Trametes* 16 (161)
		Postiaceae	*Amaropostia* 1 (2), *Calcipostia* 1 (2), *Cyanosporus* 1 (2), *Jahnoporus* 1 (1), *Oligoporus* 1 (1), *Osteina* 2 (2), *Postia* 1 (7), P*tychogaster* 1 (2), *Spongiporus* 1 (1)
		Pycnoporellaceae	*Crustoderma* 2 (4), *Pycnoporellus* 2 (8)
		Sparassidaceae	*Sparassis* 2 (5)
		Steccherinaceae	*Antrodiella* 5 (13), *Junghuhnia* 1 (5), *Metuloidea* 2 (22), *Mycorrhaphium* 1 (4), *Nigroporus* 1 (4), *Steccherinum* 5 (39)
	Russulales	Auriscalpiaceae	*Artomyces* 1 (10), *Auriscalpium* 1 (12), *Lentinellus* 7 (26)
		Bondarzewiaceae	*Bondarzewia* 3 (9), *Gloiodon* 1 (1), *Heterobasidion* 5 (7), *Laurilia* 1 (3)
		Hericiaceae	*Dentipellis* 1 (5), *Hericium* 5 (29), *Laxitextum* 1 (10)
		Peniophoraceae	*Baltazaria* 1 (1), *Gloiothele* 1 (2), *Peniophora* 8 (26), *Vararia* 1 (1)
		Stereaceae	*Conferticium* 1 (1), *Stereum* 6 (36), *Xylobolus* 4 (12)
	Xenasmatellales	Xenasmatellaceae	*Xenasmatella* 1 (1)
Dacrymycetes	Dacrymycetales	Dacrymycetaceae	*Dacryopinax* 1 (1)
Tremellomycetes	Tremellales	Phaeotremellaceae	*Phaeotremella* 1 (2)

**Table 3:** Strains from the LE-BIN collection that produce 4 lanosterol-type triterpene carboxylic acids.

Species	Strain Number	Content, mg/g, Dry Weight *			
		1	2	3	4
*Daedalea xantha*	LE-BIN 3823	5.99	0.12	3.81	0.00
*Gloeophyllum sepiarium*	LE-BIN 3412	4.55	0.59	0.94	0.40
*Gloeophyllum trabeum*	LE-BIN 0157	9.62	0.04	2.50	tr
*Inonotus obliquus*	LE-BIN 3209	4.5	tr	n/d	n/d
*Laetiporus sulphureus*	LE-BIN 3867	4.74	0.02	0.93	0.00
*Neolentinus lepideus*	LE-BIN 4332	1.27	8.83	0.29	0.55
*Oxyporus populinus*	LE-BIN 3818	0.44	0.06	0.03	0.00
*Pholiota aurivella*	LE-BIN 3929	1.59	0.24	1.00	0.23
*Pseudofavolus tenuis*	LE-BIN 4210	0.03	n/d	0.00	n/d
*Rhizomarasmius undadus*	LE-BIN 3571	0.09	tr	tr	n/d
*Sparassis crispa*	LE-BIN 2902	0.08	n/d	tr	n/d
*Stereum subtomentosum*	LE-BIN 2841	0.09	tr	tr	n/d
*Tyromyces lacteus*	LE-BIN 3990	8.19	1.40	0.07	0.01
*Vitreoporus dichrous*	LE-BIN 3131	0.07	0.02	0.01	0.01
*Vuilleminia comedens*	LE-BIN 3878	0.63	0.07	0.05	0.01

* Triterpenoid acids: eburicoic 1, trametenolic 2, dehydroeburicoic 3, dehydrotrametenolic 4.

## Data Availability

Data are contained within the article and Supplementary Materials.

## Supplementary Materials

The following supporting information can be downloaded at https://www.mdpi.com/article/10.3390/jof9121196/s1. Metadata on the express assays of oxidative and cellulolytic enzymes in the studied pure cultures are presented in the Supplementary Materials (Table S1).
