# Supplementary material for: The Conservation and Study of Macromycetes in the Komarov Botanical Institute Basidiomycetes Culture Collection—Their Taxonomical Diversity and Biotechnological Prospects
Source: J Fungi (Basel). 2023 Dec 14;9(12):1196. doi: 10.3390/jof9121196 (PMC10744906; doi:10.3390/jof9121196)
Supplement: Supplementary file 1 [file jof-09-01196-s001.zip › jof-2751907-supplementary.pdf]

Table S1

Results of the express assays of oxidative and cellulolytic enzymes in the studied pure cultures

**Strains / Species**	**GenBank accession number**	**Rot type**	**LGR[1]**	**Enzymatic activity, d (mm)**		**Origin of strains**		**References**
				**ABTS[2]**	**CMC[3]**	**Region**	**Host tree**	
AGARICALES								
Pleurotaceae								
LE-BIN 4126 *Pleurotus ostreatus*	—	WR[4]	M	17.9±1.0	21.2±1.0	Bryansk Oblast	*Acer platanoides*	1
LE-BIN 4132 *P. ostreatus*	—	WR	M	15.7±0.7	21.4±0.9	Bryansk Oblast	*Acer platanoides*	1
LE-BIN 4111 *Pleurotus pulmonarius*	—	WR	M	18.6±1.2	28.8±1.1	Bryansk Oblast	*Populus tremula*	
Pterulaceae								
LE-BIN 3587 *Radulomyces confluens*	—	WR	F	10.4±0.6	20.7±0.6	Bryansk Oblast	*Populus tremula*	1
CANTHARELLALES								
Hydnaceae								
LE-BIN 3583 *Sistotrema raduloides*	—	WR	S	n/d	25.5±0.6	Bryansk Oblast	*Populus tremula*	1
CORTICIALES								
Punctulariaceae								
LE-BIN 3603 *Punctularia strigosozonata*	—	WR	M			Bryansk Oblast	*Populus tremula*	1
GLOEOPHYLLALES								
Gloeophyllaceae								
LE-BIN 4338 *Gloeophyllum sepiarium*	—	BR[5]	S	n/d[6]	20.4±1.1	Belgorod Oblast	*Pinus sylvestris*	2
LE-BIN 4339 *G. trabeum*	—	BR	S	25.6±1.3	29.9±0.9	Belgorod Oblast	*Pyrus communis*	2
LE-BIN 4332 *Neolentinus lepideus*	—	BR	M	n/d	21.9±1.0	Belgorod Oblast	*Pinus sylvestris*	2
HYMENOCHAETALES								
Hymenochaetaceae								
LE-BIN 4785 *Fomitiporia hippophaeicola*	OQ555678	WR	M	12.2±0.7	16.5±0.2	Republic of Dagestan	*Hippophaë rhamnoides*	11
LE-BIN 4345 *Inonotus hispidus*	—	WR	M	n/d	30.8±1.5	Belgorod Oblast	*Malus domestica*	2
LE-BIN 4112 *Inonotus rheades*	—	WR	M	n/d	21.6±1.0	Bryansk Oblast	*Populus tremula*	1
LE-BIN 4344 *Phellinus alni*	—	WR	S	17.5±1.2	20.7±0.8	Kursk Oblast	*Malus domestica*	2
LE-BIN 4412 *Ph. alni*	—	WR	S	n/d	15.3±1.1	Kursk Oblast	*Malus domestica*	2
LE-BIN 4108 *Phellinus laevigatus*	—	WR	F	19.8±3.0	18.2±1.1	Bryansk Oblast	*Betula pendula*	1
LE-BIN 4340 *Phellinus pomaceus*	—	WR	M	n/d	24.4±0.7	Belgorod Oblast	*Prunus cerasus*	2
LE-BIN 4409 *Ph. pomaceus*	—	WR	M	n/d	21.1±0.8	Belgorod Oblast	*Prunus domestica*	2
LE-BIN 4410 *Ph. pomaceus*	—	WR	F	15.0±1.6	19.7±0.5	Kursk Oblast	*Prunus domestica*	2
Hymenochaetales genera incertae sedis								

LE-BIN 4405 *Trichaptum biforme*	—	WR	M	15.8±1.2	13.5±0.8	Lipetsk Oblast	*Malus domestica*	2
Oxyporaceae								
LE-BIN 3818 *Oxyporus populinus*	—	WR	M	25.1±0.8	n/d	Lipetsk Oblast	*Salix* sp.	2
Schizoporaceae								
LE-BIN 3967 *Xylodon paradoxus*	—	WR	F	26.2±0.9	19.8±1.4	Bryansk Oblast	*Populus tremula*	1
LE-BIN 3973 *X. paradoxus*	—	WR	F	28.1±1.2	20.2±1.1	Bryansk Oblast	*Populus tremula*	1
POLYPORALES								
Polyporales genera incertae sedis								
LE-BIN 3823 *Amyloporia xantha*	—	BR	M	n/d	20.9±1.6	Lipetsk Oblast	*Acer platanoides*	2
Cerrenaceae								
LE-BIN 3820 *Cerrena unicolor*	—	WR	M	15.1±1.4	n/d	Lipetsk Oblast	*Betula pendula*	2
LE-BIN 3598 *Spongipellis spumea*	—	WR	F	n/d	25.3±1.1	Bryansk Oblast	*Populus tremula*	1
Fomitopsidaceae								
LE-BIN 4109 *Fomitopsis betulina*	—	WR	F	n/d	23.6±0.9	Bryansk Oblast	*Betula pendula*	1
LE-BIN 4115 *F. betulina*	—	WR	F	n/d	21.7±1.3	Bryansk Oblast	*Betula pendula*	1
LE-BIN 3969 *Fomitopsis pinicola*	OQ053214	WR	M	29.4±1.0	22.7±1.2	Bryansk Oblast	*Populus tremula*	1
Hyphodermataceae								
LE-BIN 3596 *Hyphoderma mutatum*	—	WR	M	14.8±0.8	25.6±0.6	Bryansk Oblast	*Populus tremula*	1
LE-BIN 3813 *H. mutatum*	—	WR	M	10.8±0.7	14.4±1.1	Lipetsk Oblast	*Quercus robur*	2
LE-BIN 3974 *H. mutatum*	—	WR	M	25.5±1.5	22.3±0.9	Bryansk Oblast	*Populus tremula*	1
LE-BIN 4401 *H. mutatum*	MZ018636	WR	F	21.3±1.0	21.5±0.6	Lipetsk Oblast	*Malus domestica*	2
Incrustoporiaceae								
LE-BIN 3990 *Postia lactea*	OM033736	WR	S	n/d	42.7±1.3	Bryansk Oblast	*Alnus glutinosa*	1
Irpicaceae								
LE-BIN 3619 *Byssomerulius corium*	—	WR	M	19.1±0.4	16.6±0.7	Kursk Oblast	deciduous	2
LE-BIN 4403 *Ceriporia torpida*	—	WR	S	n/d	22.2±1.0	Lipetsk Oblast	*Malus domestica*	2
LE-BIN 4407 *C. torpida*	—	WR	F	n/d	20.3±1.0	Lipetsk Oblast	*Malus domestica*	2
LE-BIN 3978 *Gloeoporus dichrous*	—	WR	M	n/d	10.4±0.9	Bryansk Oblast	*Corylus avellana*	1
LE-BIN 4110 *G. dichrous*	—	WR	M	n/d	19.7±1.1	Bryansk Oblast	*Corylus avellana*	1
LE-BIN 3610 *Irpex lacteus*	—	WR	F	19.4±0.3	10.9±1.0	Kursk Oblast	*Corylus avellana*	2
LE-BIN 3835 *I. lacteus*	MZ018632	WR	F	20.4±0.6	22.6±1.7	Lipetsk Oblast	*Fraxinus excelsior*	2
LE-BIN 4341 *I. lacteus*	—	WR	F	15.2±0.7	21.1±0.8	Belgorod Oblast	*Prunus cerasus*	2
LE-BIN 4404 *Raduliporus aneirinus*	—	WR	M	n/d	30.2±0.4	Lipetsk Oblast	*Malus domestica*	2
LE-BIN 3607 *Trametopsis cervina*	—	WR	F	10.3±0.7	27.6±1.4	Bryansk Oblast	*Populus tremula*	1
Laricifomitaceae								
LE-BIN 3992 *Rhodonia placenta*	—	BR	F	24.9±0.7	19.8±1.4	Bryansk Oblast	*Pinus sylvestris*	1
Meruliaceae								

LE-BIN 4002 *Aurantiporus fissilis*	—	WR	F	n/d	33.4±1.0	Bryansk Oblast	*Populus tremula*	1
LE-BIN 4119 *A. fissilis*	—	WR	M	26.0±1.2	21.8±0.9	Bryansk Oblast	*Acer platanoides*	1
LE-BIN 4127 *Phlebia rufa*	—	WR	M	20.9±1.9	n/d	Bryansk Oblast	*Populus tremula*	1
LE-BIN 4128 *P. rufa*	—	WR	M	20.2±2.1	26.1±0.9	Bryansk Oblast	*Acer platanoides*	1
LE-BIN 3591 *Phlebia tremellosa*	—	WR	F	18.4±0.6	20.9±0.7	Bryansk Oblast	*Tilia cordata*	1
LE-BIN 2138 *Sarcodontia crocea*	—	WR	S	15.0±0	22.5±0,6	Rostov Oblast	*Malus domestica*	8
LE-BIN 4342 *S. crocea*	MW042103	WR	S	29.9±2.2	n/d	Belgorod Oblast	*Malus domestica*	7, 8
LE-BIN 4343 *S. crocea*	MW042104	WR	M	31.2±0.9	21.5±0,6	Belgorod Oblast	*Malus domestica*	7, 8
LE-BIN 4346 *S. crocea*	—	WR	S	31.8±1.1	19.8±0.7	Belgorod Oblast	*Malus domestica*	7, 8
LE-BIN 4350 *S. crocea*	MW042105	WR	M	34.8±0.8	18.9±0.5	Belgorod Oblast	*Malus domestica*	7
LE-BIN 4355 *S. crocea*	MW042106	WR	M	28.9±1.4	20.9±1.1	Oryol Oblast	*Malus domestica*	7, 8, 9
LE-BIN 4365 *S. crocea*	MW042107	WR	S	36.3±1.1	22.1±1.1	Oryol Oblast	*Malus domestica*	7, 8
LE-BIN 4367 *S. crocea*	MW042108	WR	S	27.9±1.2	12.9±1.0	Oryol Oblast	*Malus domestica*	7, 8
LE-BIN 4378 *S. crocea*	MW042109	WR	S	23.0±0.9	n/d	Oryol Oblast	*Malus domestica*	7
LE-BIN 4382 *S. crocea*	—	WR	M	28.6±1,5	21.6±0,6	Oryol Oblast	*Malus domestica*	7, 8
LE-BIN 4398 *S. crocea*	—	WR	S	19.0±1.6	12.0±0.9	Lipetsk Oblast	*Malus domestica*	2
LE-BIN 4399 *S. crocea*	—	WR	M	16.7±1.4	11.3±1.0	Lipetsk Oblast	*Malus domestica*	2
LE-BIN 4400 *S. crocea*	—	WR	S	26.3±1.4	13.3±1.0	Lipetsk Oblast	*Malus domestica*	2
LE-BIN 4402 *S. crocea*	—	WR	S	22.7±1.2	14.3±1.0	Lipetsk Oblast	*Malus domestica*	2
LE-BIN 4406 *S. crocea*	—	WR	S	24.5±1.2	16.2±1.5	Lipetsk Oblast	*Malus domestica*	2
LE-BIN 4408 *S. crocea*	—	WR	S	29.2±3.3	15.3±1.2	Lipetsk Oblast	*Malus domestica*	2
LE-BIN 4411 *S. crocea*	—	WR	S	30.7±0.8	13.0±1.1	Kursk Oblast	*Malus domestica*	2
Phanerochaetaceae								
LE-BIN 3996 *Bjerkandera adusta*	—	WR	F	n/d	25.7±1.0	Bryansk Oblast	*Populus tremula*	1
LE-BIN 4107 *Bjerkandera fumosa*	—	WR	F	n/d	24.8±0.8	Bryansk Oblast	*Alnus glutinosa*	1
LE-BIN 3811 *Porostereum spadiceum*	—	WR	F	n/d	20.5±1.0	Lipetsk Oblast	*Quercus robur*	2
Podoscyphaceae								
LE-BIN 3616 *Abortiporus biennis*	—	WR	M	20.0±1.1	n/d	Kursk Oblast	*Salix* sp.	2
Polyporaceae								
LE-BIN 3615 *Daedaleopsis confragosa*	—	WR	F	24.7±1.6	20.1±0.3	Kursk Oblast	*Populus nigra*	2
LE-BIN 3616 *D. confragosa*	—	WR	M	22.5±2.1	18.8±1.4	Lipetsk Oblast	*Betula pendula*	2
LE-BIN 3624 *D. confragosa*	—	WR	F	17.8±0.3	11.0±0.4	Kursk Oblast	*Prunus padus*	2
LE-BIN 3625 *D. confragosa*	—	WR	M	22.8±0.8	19.2±0.2	Kursk Oblast	*Salix* sp.	2
LE-BIN 3989 *D. confragosa*	—	WR	M	18.3±0.8	24.9±0.8	Bryansk Oblast	*Populus tremula*	1
LE-BIN 3828 *Funalia trogii*	MZ018630	WR	F	28.7±2.9	22.9±1.3	Lipetsk Oblast	*Populus tremula*	2
LE-BIN 3582 *Ganoderma applanatum*	—	WR	F	17.4±2.0	22.8±0.8	Bryansk Oblast	*Populus tremula*	1
LE-BIN 4003 *G. applanatum*	—	WR	F	n/d	22.2±0.8	Bryansk Oblast	*Quercus robur*	1

LE-BIN 3620 *Ganoderma lucidum*	—	WR	S	23.8±0.9	20.9±1.6	Kursk Oblast	*Quercus robur*	2
LE-BIN 4333 *Lentinus arcularius*	MZ018633	WR	M	27.8±0.9	17.2±0.9	Belgorod Oblast	*Quercus robur*	2
LE-BIN 4334 *Neofavolus alveolaris*	—	WR	M	n/d	21.3±0.9	Belgorod Oblast	*Acer negundo*	2
LE-BIN 4122 *Tramates hirsuta*	—	WR	F	26.9±0.9	31.6±1.0	Bryansk Oblast	*Acer platanoides*	1
LE-BIN 4124 *T. hirsuta*	—	WR	M	25.7±1.5	30.0±0.8	Bryansk Oblast	*Corylus avellana*	1
LE-BIN 3578 *Trametes gibbosa*	—	WR	F	14.9±0.7	23.7±0.9	Bryansk Oblast	*Quercus robur*	1
LE-BIN 4123 *Trametes ochracea*	—	WR	F	24.1±1.0	31.1±0.9	Bryansk Oblast	*Acer platanoides*	1
LE-BIN 3995 *Trametes pubescens*	—	WR	F	13.6±1.0	n/d	Bryansk Oblast	*Betula pendula*	1
LE-BIN 4335 *Trametes versicolor*	MZ018634	WR	F	26.2±0.9	28.8±0.8	Belgorod Oblast	*Salix caprea*	2
Sparassidaceae								
LE-BIN 4114 *Pycnoporellus fulgens*	—	WR	M	n/d	19.4±0.7	Bryansk Oblast	*Picea abies*	1
Steccherinaceae								
LE-BIN 3832 *Etheirodon fimbriatum*	—	WR	S	n/d	28.0±0.6	Lipetsk Oblast	*Acer platanoides*	2
LE-BIN 3623 *Junghuhnia nitida*	MZ018626	WR	S	27.8±0.4	n/d	Kursk Oblast	*Prunus padus*	2
LE-BIN 3819 *J. nitida*	MZ018629	WR	M	24.6±2.2	17.7±0.9	Lipetsk Oblast	*Salix* sp.	2
LE-BIN 3576 *Metuloidea fragrans*	—	WR	S	25.6±1.4	15.9±0.7	Bryansk Oblast	*Corylus avellana*	1
LE-BIN 3608 *M. fragrans*	MZ018625	WR	S	27.6±1.4	11.5±0.8	Kursk Oblast	*Alnus glutinosa*	2
LE-BIN 3609 *M. fragrans*	—	WR	S	29.3±1.8	15.8±0.5	Kursk Oblast	*Corylus avellana*	2
LE-BIN 3612 *M. fragrans*	—	WR	S	26.2±0.5	20.5±0.3	Kursk Oblast	*Prunus padus*	2
LE-BIN 3825 *M. fragrans*	—	WR	S	29.7±2.9	25.0±2.0	Lipetsk Oblast	*Corylus avellana*	2
LE-BIN 3830 *M. fragrans*	—	WR	S	31.3±3.0	26.2±2.2	Lipetsk Oblast	*Betula pendula*	2
LE-BIN 3834 *M. fragrans*	—	WR	S	27.0±1.1	22.8±1.8	Lipetsk Oblast	*Prunus padus*	2
LE-BIN 3972 *M. fragrans*	—	WR	M	25.3±2.6	24.6±1.9	Bryansk Oblast	*Corylus avellana*	1
LE-BIN 3976 *M. fragrans*	—	WR	M	23.1±0.9	23.0±0.5	Bryansk Oblast	*Betula pendula*	1
LE-BIN 3980 *M. fragrans*	—	WR	M	27.0±1.1	25.5±0.8	Bryansk Oblast	*Populus tremula*	1
LE-BIN 3982 *M. fragrans*	—	WR	M	19.7±1.6	27.6±1.2	Bryansk Oblast	*Corylus avellana*	1
LE-BIN 3983 *M. fragrans*	—	WR	M	15.7±0.9	24.6±1.0	Bryansk Oblast	*Corylus avellana*	1
LE-BIN 3991 *M. fragrans*	—	WR	S	26.4±1.3	22.9±1.0	Bryansk Oblast	*Corylus avellana*	1
LE-BIN 4000 *M. fragrans*	—	WR	S	25.3±1.1	22.8±1.2	Bryansk Oblast	*Betula pendula*	1
LE-BIN 4001 *M. fragrans*	—	WR	M	30.3±1.8	25.4±1.3	Bryansk Oblast	*Corylus avellana*	1
LE-BIN 3826 *Steccherinum bourdotii*	MG711809	WR	M	33.8±3.3	17.2±0.4	Lipetsk Oblast	*Quercus robur*	2
LE-BIN 3577 *Steccherinum ochraceum*	—	WR	S	26.6±1.2	13.5±0.5	Bryansk Oblast	*Corylus avellana*	1
LE-BIN 3611 *S. ochraceum*	KY321182 / MG711808	WR	M	31.8±0.3	14.4±0.8	Kursk Oblast	*Corylus avellana*	2
LE-BIN 3617 *S. ochraceum*	KY321180 / MG609023	WR	M	29.4±0.5	20.2±1.4	Kursk Oblast	*Corylus avellana*	2

LE-BIN 3622 *S. ochraceum*	KY321181 / MG609022	WR	M	25.0±0.3	n/d	Kursk Oblast	*Populus tremula*	2
LE-BIN 3824 *S. ochraceum*	MG609021	WR	S	30.0±1.4	14.9±1.1	Lipetsk Oblast	*Betula pendula*	2
LE-BIN 3827 *S. ochraceum*	MG609031	WR	S	36.3±3.2	19.3±1.7	Lipetsk Oblast	*Corylus avellana*	2
LE-BIN 3836 *S. ochraceum*	MG609020	WR	M	42.6±3.9	26.1±1.6	Lipetsk Oblast	*Acer platanoides*	2
LE-BIN 3977 *S. ochraceum*	—	WR	M	32.2±0.8	30.3±1.2	Bryansk Oblast	*Corylus avellana*	1
LE-BIN 3981 *S. ochraceum*	—	WR	S	32.5±0.7	20.3±0.8	Bryansk Oblast	*Corylus avellana*	1
LE-BIN 3984 *S. ochraceum*	—	WR	F	30.4±0.9	29.4±1.4	Bryansk Oblast	*Corylus avellana*	1
LE-BIN 3985 *S. ochraceum*	—	WR	S	16.7±0.9	20.9±1.2	Bryansk Oblast	*Quercus robur*	1
LE-BIN 4005 *S. ochraceum*	—	WR	S	33.1±1.2	28.2±1.7	Bryansk Oblast	*Corylus avellana*	1
RUSSULALES								
Hericiaceae								
LE-BIN 3594 *Hericium coralloides*	—	WR	S	18.5±1.2	12.1±0.6	Bryansk Oblast	*Populus tremula*	1
Peniophoraceae								
LE-BIN 3815 *Peniophora cinerea*	MZ018628	WR	M	16.2±0.8	22.1±1.9	Lipetsk Oblast	*Sorbus aucuparia*	2
LE-BIN 4336 *P. cinerea*	MZ018635	WR	M	27.2±0.9	22.8±0.6	Belgorod Oblast	*Pyrus communis*	2
LE-BIN 4337 *P. incarnata*	—	WR	M	22.9±2.1	29.5±1.0	Belgorod Oblast	*Prunus armeniaca*	2
LE-BIN 3814 *Peniophora quercina*	MZ018627	WR	M	21.6±2.1	28.9±2.4	Lipetsk Oblast	*Quercus robur*	2
LE-BIN 3833 *P. quercina*	MZ018631	WR	M	20.9±1.6	26.2±0.8	Lipetsk Oblast	*Quercus robur*	2
Stereaceae								
LE-BIN 3618 *Stereum* sp.	—	WR	M	13.8±0.8	17.3±0.6	Kursk Oblast	undefined wood	2
LE-BIN 3593 *Xylobolus frustulatus*	—	WR	S	n/d	22.2±0.6	Bryansk Oblast	*Quercus robur*	1
LE-BIN 4125 *X. frustulatus*	—	WR	M	n/d	21.6±0.6	Bryansk Oblast	*Quercus robur*	1

[1]LGR – linear growth rate (S – slow, M – medium, F – fast); [2]ABTS – 2,2′-azino-bis 3-ethylbenzothiazoline-6-sulfonic acid; [3]CMC – carboxymethyl cellulose; [4]WR – white rot; [5]BR – brown rot; [6]n/d – not detected.

References

1. Shakhova, N.; Volobuev, S. Revealing new active and biotechnologically perspective producers of oxidative and cellulolytic enzymes among pure cultures of xylotrophic Agaricomycetes from the Southern Non-Chernozem zone of the European part of Russia. Curr. Res. Environ. Appl. Mycol. J. Fungal Biol. 2020a, 10(1), 113–119. https://doi. org/10.5943/cream/10/1/12

2. Volobuev, S.; Shakhova, N. Towards the Discovery of Active Lignocellulolytic Enzyme Producers: A Screening Study of Xylotrophic Macrofungi from the Central Russian Upland. Iranian Journal of Science and Technology, Transactions A: Science. 2022, 46(1), 91–100. https://doi.org/10.1007/s40995-021-01245-7

3. Glazunova, O. A.; Shakhova, N. V.; Psurtseva, N. V.; Moiseenko, K. V.; Kleimenov, S. Y.; Fedorova T. V. White-rot basidiomycetes Junghuhnia nitida and Steccherinum bourdotii: Oxidative potential and laccase properties in comparison with Trametes hirsuta and Coriolopsis caperata. PLoS One. 2018, 13(6), e0197667. https://doi.org/10.1371/journal.pone.0197667

4. Moiseenko, K.; Glazunova, O.; Shakhova, N.; Savinova, O.; Vasina, D.; Tyazhelova, T.; Psurtseva, N.; Fedorova, T. Data on the genome analysis of the wood-rotting fungus Steccherinum ochraceum LE-BIN 3174. Data in brief. 2020, 29, 105169. https://doi.org/10.1016/j.dib.2020.105169

5. Moiseenko, K. V. Glazunova, O. A.; Shakhova, N. V.; Savinova, O. S.; Vasina, D. V.; Tyazhelova, T. V.; Psurtseva, N.V.; Fedorova, T. V. Fungal adaptation to the advanced stages of wood decomposition: Insights from the Steccherinum ochraceum. Microorganisms. 2019, 7(11), 527–543. https://doi.org/10.3390/microorganisms7110527

6. Glazunova, O. A.; Moiseenko, K. V.; Shakhova, N. V.; Psurtseva, N. V.; Fedorova, T. V. Biodegradation potential of Steccherinum ochraceum: growth on different wood types and preliminary evaluation of enzymatic activities. KnE Life Sciences. 2022, 7(10), 207–215. https://doi.org/10.18502/kls.v7i1.10123

7. Shakhova, N. V.; Volobuev, S. V. Culture characteristics and enzymatic activity of Sarcodontia crocea (Basidiomycota) strains collected from the Central Russian Upland. Mycology and Phytopathology. 2020b, 54 (6), 446–451. https://doi.org/10.31857/S0026364820060100

8. Volobuev, S. V.; Shakhova N. V. A comparative study on growth and lignocellulolytic activity of nine Sarcodontia crocea strains in four different media. Diversity of plant world. 2020, 3(6), 45–54. https://doi.org/10.22281/2686-9713-2020-3-45-54

9. Whaley, A. K.; Ponkratova, A. O.; Orlova, A. A.; Volobuev, S. V.; Shakhova, N, V.; Serebryakov, E. B.; Smirnov, S. N.; Pustovit, N. V.; Kraeva, L. A.; Luzhanin V. G.; New benzoquinone pigments from the hydnoid fungus Sarcodontia setosa and their biosynthetic relationship. Natural Product Research. 2023a, 1–10. https://doi.org/10.1080/14786419.2023.2195176

10. Whaley, A. O.; Whaley, A. K.; Orlova, A. A.; Volobuev, S. V.; Shakhova, N, V.; Smirnov, S. N.; Pustovit, N. V.; Kraeva, Polyacetylene derivatives from the Polypore fungus (Fistulina hepatica, Agaricomycetes) and their antimicrobial activity. International Journal of Medicinal Mushrooms. 2023b, 25(12), 43−53.

11. Shakhova, N.; Volobuev, S. Cultural and enzymatic activity studies of a pathogenic wood-decaying fungus Fomitiporia hippophaeicola (Hymenochaetales, Basidiomycota), recollected in the Eastern Caucasus. Arch Microbiol 205. 2023, Article number: 249. https://doi.org/10.1007/s00203-023-03587-9
